# Single-stranded pre-methylated 5mC adapters uncover the methylation profile of plasma ultrashort Single-stranded cell-free DNA

**DOI:** 10.1093/nar/gkae276

**Published:** 2024-05-27

**Authors:** Jordan C Cheng, Neeti Swarup, Marco Morselli, Wei-Lun Huang, Mohammad Aziz, Christa Caggiano, Misagh Kordi, Abhijit A Patel, David Chia, Yong Kim, Feng Li, Fang Wei, Noah Zaitlen, Kostyantyn Krysan, Steve Dubinett, Matteo Pellegrini, David T W Wong

**Affiliations:** School of Dentistry, University of California, Los Angeles, Los Angeles, CA 90095, USA; School of Dentistry, University of California, Los Angeles, Los Angeles, CA 90095, USA; Department of Molecular, Cell, and Developmental Biology, Life Sciences Division, University of California, Los Angeles, Los Angeles, CA 90095, USA; Department of Chemistry, Life Sciences and Environmental Sustainability, University of Parma, Parma, Italy; Center of Applied Nanomedicine, National Cheng Kung University, Tainan, Taiwan; School of Dentistry, University of California, Los Angeles, Los Angeles, CA 90095, USA; Department of Computational Medicine, University of California Los Angeles, Los Angeles, CA, USA; School of Dentistry, University of California, Los Angeles, Los Angeles, CA 90095, USA; Department of Therapeutic Radiology, Yale University, New Haven, CT, USA; Department of Pathology, David Geffen School of Medicine, University of California, Los Angeles, Los Angeles, CA 90095, USA; School of Dentistry, University of California, Los Angeles, Los Angeles, CA 90095, USA; School of Dentistry, University of California, Los Angeles, Los Angeles, CA 90095, USA; School of Dentistry, University of California, Los Angeles, Los Angeles, CA 90095, USA; Department of Computational Medicine, University of California Los Angeles, Los Angeles, CA, USA; Department of Medicine, David Geffen School of Medicine at UCLA, Los Angeles, CA, USA; Department of Medicine, David Geffen School of Medicine at UCLA, Los Angeles, CA, USA; Department of Molecular, Cell, and Developmental Biology, Life Sciences Division, University of California, Los Angeles, Los Angeles, CA 90095, USA; School of Dentistry, University of California, Los Angeles, Los Angeles, CA 90095, USA

## Abstract

Whole-genome bisulfite sequencing (BS-Seq) measures cytosine methylation changes at single-base resolution and can be used to profile cell-free DNA (cfDNA). In plasma, ultrashort single-stranded cfDNA (uscfDNA, ∼50 nt) has been identified together with 167 bp double-stranded mononucleosomal cell-free DNA (mncfDNA). However, the methylation profile of uscfDNA has not been described. Conventional BS-Seq workflows may not be helpful because bisulfite conversion degrades larger DNA into smaller fragments, leading to erroneous categorization as uscfDNA. We describe the ‘5mCAdpBS-Seq’ workflow in which pre-methylated 5mC (5-methylcytosine) single-stranded adapters are ligated to heat-denatured cfDNA before bisulfite conversion. This method retains only DNA fragments that are unaltered by bisulfite treatment, resulting in less biased uscfDNA methylation analysis. Using 5mCAdpBS-Seq, uscfDNA had lower levels of DNA methylation (∼15%) compared to mncfDNA and was enriched in promoters and CpG islands. Hypomethylated uscfDNA fragments were enriched in upstream transcription start sites (TSSs), and the intensity of enrichment was correlated with expressed genes of hemopoietic cells. Using tissue-of-origin deconvolution, we inferred that uscfDNA is derived primarily from eosinophils, neutrophils, and monocytes. As proof-of-principle, we show that characteristics of the methylation profile of uscfDNA can distinguish non-small cell lung carcinoma from non-cancer samples. The 5mCAdpBS-Seq workflow is recommended for any cfDNA methylation-based investigations.

## Graphical Abstract

**Graphical Abstract:**
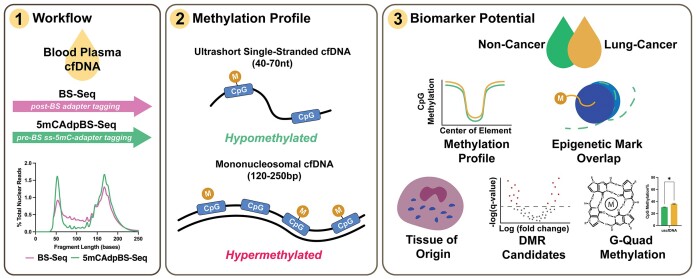

## Introduction

Cancer contributes to substantial morbidity and mortality worldwide, with 19.3 million new cancer cases and 10.0 million deaths in 2020 (1). Early cancer detection remains the best strategy for improving patient prognosis (2). A liquid biopsy strategy in which biomolecules are analyzed within biofluids can provide non-invasive, easily repeated, and real-time insights into a patient's tumor burden and treatment response (3). Although many biomolecules (e.g. circulating tumor cells, exosomes, proteins, RNAs, or metabolites) are viable for liquid biopsy, there has been a focus in prior studies on cell-free DNA (cfDNA). Examining methylation characteristics inherent within cfDNA fragments is a promising approach (4,5). Dysregulated epigenetic control in tumor cells contributes to tumorigenesis and genetic instability. Both global hypomethylation (6) and regional hypermethylation (7) in cfDNA are hallmarks of cancer cells. Approximately 60–80% of the 28 million CpG sites are methylated in humans (8), and any deviations in the cfDNA global profile could suggest aberrant methylation originating from cells other than those of hematopoietic origin, which contribute to the majority of cfDNA (9). In addition, DNA methylation patterns are more consistent among the cancer cells within a tumor, unlike somatic mutations, which may be present at low frequencies in a tumor (10).

The observed cytosine methylation, fragmentation, and strandedness of cfDNA are influenced by both the biological origins and laboratory workflow. We have previously shown that preprocessing cfDNA using the broad-range cell-free DNA sequencing pipeline (BRcfDNA-Seq) reveals the presence of ultrashort single-stranded cell-free DNA (uscfDNA) of approximately 50 nt in addition to conventionally reported ∼167 bp mononucleosomal-cell free DNA (mncfDNA) (11,12). BRcfDNA-Seq uses enhanced isopropanol extraction coupled with single-stranded DNA library preparation to capture and incorporate short single-stranded or nicked cfDNA fragments routinely lost when using double-stranded library kits. This ultrashort population has also been observed by other groups using similar extraction and library protocols (13–16). Although mncfDNA has been thoroughly investigated as a cfDNA biomarker for tissue-of-origin deconvolution and cancer detection (10,17), the epigenetic properties of uscfDNA have yet to be examined.

Whole-genome bisulfite sequencing (BS-Seq) is a bisulfite-based sequencing technique that indicates the methylation state of all cytosines in a DNA sample at the resolution of a single base pair (17). Alternative methods, such as reduced representation bisulfite sequencing (RRBS) (18) and methylated DNA immunoprecipitation (MeDIP-Seq) (19), have been used to profile cfDNA but only provide information on ∼10% of the whole genome. In an unexplored biological context, such as uscfDNA, it may be important to gather a genome-wide measure of methylation before focusing on specific regions of interest.

For low-input samples typical of cfDNA, BS-Seq requires treatment with sodium bisulfite prior to the ligation of adapters and subsequent amplification. One drawback of bisulfite conversion is that it damages DNA, resulting in the creation of artificially smaller fragments (17). The degree of degradation has been reported to be inversely proportional to the fragment size, with large genomic DNA being the most susceptible (20,21). It has been reported that fragments up to 131 bp will typically undergo a 20% loss due to degradation, whereas those that are approximately 62 bp in size will experience a 10% loss (20,22). The impact of artificially generated ultrashort fragments from bisulfite degradation should be minimized, as many cfDNA studies are contingent on the accurate measurement of the size profiles of fragments. Thus, avoiding bisulfite-induced degradation reduces the potential masking of the actual signal from natively ultrashort cfDNA fragments.

In this report, we propose an approach to reduce the inclusion of bisulfite-induced degradation in the final library by ligating pre-methylated single-stranded adapters to heat-denatured cfDNA fragments prior to bisulfite conversion (5mCAdpBS-Seq protocol). Although pre-methylated adapter techniques have been introduced previously (23,24), the single-stranded version of the adapter has not been used for studying single-stranded cfDNA (11). We show that this method provides a more comprehensive characterization of uscfDNA and mncfDNA compared to samples that undergo BS-Seq. Using this technique, we were able to profile the methylation level of cytosines in both uscfDNA and mncfDNA for the first time. As a proof of concept, we show that various methylation features of uscfDNA can be used as novel biomarkers for cancer detection by analyzing a small cohort of plasma samples from late-stage non-small cell lung carcinoma (NSCLC) patients compared to non-cancer controls.

## Materials and methods

### Clinical samples

Plasma from healthy donors (Table 1) was purchased from Innovative Research (IPLASK2E10ML) in K2EDTA tubes. According to vendor instructions, whole blood was spun at 5000 × g for 15 min and the plasma removed using a plasma extractor.

**Table 1.** Samples in study

Paired BS-Seq and 5mCAdpBS-Seq				
Number	Lot number	Age of subject, years	Sex	
1	666	38	M	
2	668	52	M	
3	681	18	M	
4	698	26	F	
5	700	35	F	
**NSCLC samples**				
**Number**	**Lot number**	**Age of patient, years**	**Stage**	**Sex**
1	120E	47	3A	F
2	147E	75	4	F
3	161E	79	3B	F
4	231E	62	3A	M

Plasma from late-stage NSCLC patients (Table 1) was obtained from UCLA in an NIH-funded project (4UH3CA206126-03: Advancing EFIRM-Liquid Biopsy (eLB) to a CLIA-Certified Laboratory Developed Test (eLB-LDT) for Detection of Actionable *EGFR* mutations in NSCLC Patients, IRB#17-000997). Biopsy specimens were examined histologically and staged with the American Joint Committee on Cancer (AJCC) TNM system (25).

### Lambda DNA control restriction enzyme reactions

A combination of restriction enzymes was used to create reproducible lambda controls with consistent fragment patterns that were not influenced by the duration of enzyme treatment. This reaction ensured that fragments would be present in the size range of interest (25–100 bases). For all reactions, 1.5 μl (1 μg) of unmethylated lambda phage genomic DNA (Promega, D1521) was used. After the restriction enzyme reaction described below, the DNA was purified by combining 20 μl of the reaction mixture with 60 μl of SPRI-select beads and 60 μl of 100% isopropanol and incubated for 10 min. The tube was placed on a magnetic rack for 5 min to allow the beads to separate. The supernatant was discarded, and the beads were washed twice with 200 μl of 80% ethanol. After removing the ethanol, the beads were air dried for 10 min. The bead pellet was resuspended in 20 μl of elution buffer (QIAGEN, 19086), and the tubes were placed on the magnetic rack for 2 min. The clear supernatant was transferred into a new tube. The purified digested lambda products were combined and diluted to produce a mixture with a final concentration of 50 pg/μl.

CviKL restriction enzyme: 1.5 μl of lambda DNA was combined with 2 μl (10×) rCutSmart Buffer, 1 μl CviKL enzyme (NEB, R0710S), and 15.5 μl of nuclease-free H_2_O. The mixture was incubated at 25°C for 60 min, followed by enzyme inactivation at 65°C for 20 min.

NlaIII restriction enzyme: 1.5 μl of lambda DNA was combined with 2 μl (10×) rCutSmart Buffer, 1 μl Nlalll (NEB, R0125S), and 15.5 μl H_2_O. The mixture was incubated at 37°C for 15 min, and then the enzyme was deactivated by heating it to 65°C for 20 min.

AluI restriction enzyme: 1.5 μl of lambda DNA was combined with 2 μl (10×) rCutSmart Buffer, Alul (NEB, R0137S), and 15.5 μl H_2_O. The mixture was incubated at 37°C for 60 min, and then the enzyme was deactivated by heating it to 65°C for 20 min.

### Nucleic acid extraction

cfDNA was extracted from plasma using the QIAmp Circulating Nucleic Acid Kit (Qiagen, 55114) following the manufacturer's protocol ‘Purification of Circulating microRNA from 1 ml of Plasma’ (QiaM). For BRcfDNA-Seq (11), cfDNA was extracted from 1 ml of plasma. For the methylation pipeline, cfDNA was extracted from 2 ml of plasma. Proteinase K digestion was carried out as instructed. Carrier RNA was not used for BRcfDNA-Seq, BS-Seq and 5mCAdpBS-Seq. The ATL Lysis Buffer (Qiagen, 19076) was used as indicated in the microRNA protocol. The final elution volume for all protocols was 20 μl.

### BRcfDNA-Seq library preparation

Single-stranded DNA library preparation was performed using the SRSLY^TM^ PicoPlus DNA NGS Library Preparation Base Kit with the SRSLY 12 UMI-UDI primer set and unique molecular identifier (UMI) add-on reagents, and purified using Clarefy purification beads and the low molecular weight protocol (Claret Bioscience, CBS-K250B-24, CBS-UM-24, CBS-UR-24, CBS-BD-24). As an optimized method for specifically measuring uscfDNA is not yet available, 18 μl of extracted cfDNA was used as input and heat-denatured as instructed. In experiments including digested lambda DNA, the library preparation was spiked with 50 pg. The index PCR was performed as specified in the manual for 11 cycles. All bead clean-up steps followed the low molecular weight retention purification protocol (26). Specifically, after adapter ligation, the 50 μl reaction was combined with 48 μl of water, 12 μl of 100% isopropanol, and 65.2 μl of Clarefy beads. After the UMI extension, the 40 μl reaction was combined with 80 μl of Clarefy beads. After the index PCR, the 50 μl reaction was combined with 75 μl of Clarefy beads.

### BS-Seq library preparation

First, for the BS-Seq protocol (post-bisulfite adapter tagging), 20 μl of extracted DNA underwent bisulfite conversion using the Zymo Research DNA Methylation Lightning kit (Zymo Research, D5030) with an elution volume of 20 μl. Subsequently, single-stranded libraries were constructed as described above. During the final index PCR, the Index PCR Master Mix was substituted with the Kapa HIFI HotStart Uracil + ReadyMix. The bisulfite PCR protocol was as follows: 98°C for 3 min; 11 cycles of 98°C for 30 s, 60°C for 30 s, 72°C for 1 minute; 72°C for 1 min; then hold at 12°C. All bead clean-up steps followed the low molecular weight retention purification protocol (26). Specifically, after adapter ligation, the 50 μl reaction was combined with 48 μl of water, 12 μl of 100% isopropanol, and 65.2 μl of Clarefy beads. After the UMI extension, the 40 μl reaction was combined with 80 μl of Clarefy beads. After the index PCR, the 50 μl reaction was combined with 75 μl of Clarefy beads.

### 5mCAdpBS-Seq library preparation

The first step of the single-stranded library preparation (pre-methylated single-stranded adapter ligation) was performed on extracted cfDNA prior to bisulfite conversion. Custom 5mC-protected SRSLY adapters were provided by Claret Bioscience and are identical to those found in the regular SRSLY kit, with the exception that all cytosine residues on the adapter strands of the duplexed splint adapters are pre-methylated (5mC). The adapter sequences were as follows: 5′-Adapter (5′-A5mCA 5mCT5mC TTT 5mC5mC5mC TA5mC A5mCG A5mCG 5mCT5mC TT5mC 5mCGA T5mCT-3′) and 3′ Adapter (5′-AGA T5mCG GAA GAG 5mCA5mC A5mCG T5mCT GAA 5mCT5mC 5mCAG T5mCA 5mC-3′). These were used in place of the regular adapters in the adapter ligation step and, after bead clean-up, resuspended to 20 μl. Then 20 μl of adapter-ligated DNA underwent bisulfite conversion using the Zymo Research DNA Methylation Lightning kit (Zymogen, D5030) with an elution volume of 15 μl in the UMI-UDI step of the single-strand library preparation protocol. The remaining steps (Addition of UMI by Primer extension and Index PCR) were performed as described for BRcfDNA-Seq library preparation above. During the final index PCR, the Index PCR Master Mix was substituted with the Kapa HIFI HotStart Uracil + ReadyMix. The PCR protocol was as follows: 98°C for 3 min; 11 cycles of 98°C for 30 s, 60°C for 30 s, 72°C for 1 min; 72°C for 1 min; and hold at 12°C. All bead clean-up steps followed the low molecular weight retention purification protocol (26). Specifically, after adapter ligation, the 50 μl reaction was combined with 48 μl of water, 12 μl of 100% isopropanol, and 65.2 μl of Clarefy beads. After the UMI extension, the 40 μl reaction was combined with 80 μl of Clarefy beads. After the index PCR, the 50 μl reaction was combined with 75 μl of Clarefy beads.

### Final library concentration and quality control

Library concentrations were measured using the Qubit Fluorometer (ThermoFisher Scientific, Q33327), and quality was assessed using the Tapestation 4200 using D1000 High-Sensitivity Assay (Agilent Technologies, 5067–5584). Samples were pooled to a final molarity of 5 nM.

### Sequencing

Pooled libraries were sequenced 150 bp × 2 on NovaSeq6000 in either an SP or S1 flow cell, aiming at 40 million reads per sample.

### Data analysis

Paired reads were merged into single-end reads using BBMerge (27) to obtain one fastq file per sample. Each fastq file was trimmed with fastp using the adapter sequence AGATCGGAAGAGCACACGTCTGAACTCCAGTCAC (r1) and AGATCGGAAGAGCGTCGTGTAGGGAAAGAGTGT (r2) and filtered for a Phred score > 15 (28). Standard (unconverted) and BS-treated libraries were aligned against the combined human (GenBank: GCA_000001305.2, GRCh38) and lambda phage (GenBank: J02459.1) reference genomes using BWA-mem (29) and BSBolt's default setting (30), respectively. Sequence reads were demultiplexed using the SRSLYumi python package (SRSLYumi 0.4 version, Claret Bioscience). The duplicated reads were removed using the Picard Toolkit (http://broadinstitute.github.io/picard/) with VALIDATION_STRINGENCY set as LENIENT and REMOVE_DUPLICATES as TRUE. Soft and hard clipped reads were removed using samtools (version 1.9). Quality control assessment was performed with Qualimap Version 2.2.2c (31). Samtools View was also used to isolate reads from the mitochondrial DNA. Each of the bam files aligning to human, mitochondria, and lambda phage was binned in increments of 10 bases from 20 to 200 using alignmentSieve (deepTools 3.5.0) (32).

### CpG and non-CpG methylation%

BSBolt CallMethylation was used on each 10-base bin to determine the %CG methylation and %CHH methylation. MapQ scores were calculated from each size bin by Qualimap (version 2.2.2c) (31).

### G-Quad signatures

G-Quad percentage was calculated by first converting binned bam files to bed files using bamtobed (bedtools version 2.18) and then from bed to fasta files using getfasta (bedtools version 2.18) (33). The software fastaRegexFinder.py was used to analyze the sequences in the reads (https://github.com/dariober/bioinformatics-cafe/tree/master/fastaRegexFinder). In general, this python pipeline examines whether the sequences contain this pattern in the equation: ‘([gG]{3,}\w{1,7}){3,}[gG]{3,}’. This translates to identifying three or more G nucleotides followed by 1–7 of any other bases and must be repeated three or more times and end with three or more Gs. The G-Quad counts were divided by the total read counts to identify the G-Quad percentage. A normalized ratio was calculated by dividing the read counts by the median value of the bin length (e.g. 20–29 is 25). Only primary fragments that contained G-Quad sequences were counted (e.g. complementary sequences that contained G-Quads were excluded). The coordinates of the G-Quad sequences were used to generate G-Quad Only bam files for calling the CpG methylation percentage.

### Linear correlation analysis

The bam files were split into genomic bins of 100k bases along the genome [e.g. Chr1: 1–100 000 for two *in-silico* categories, uscfDNA (40–70 bases) and mncfDNA (120–250 bases)]. The percent total coverage was calculated for each bin, and then the linear correlation was calculated by comparing the signal from each genomic bin position in Graphpad Prism 9. Both samples processed with BS-Seq and 5mCAdpBS-Seq were compared to the paired equivalent BRcfDNA-Seq sample.

### Genome-wide ideogram

Bed files containing the location of each CpG site were split into genomic bins of 1 million bases along the genome. Karyograms were self-normalized so that the legend reflects the intrasample dynamic range. Ideograms were constructed from the average of five samples, showing the CpG site frequency for each 1 million-base bin using the RIdeogram R package (34).

### CpG intersection positions with genomic elements

CGmap files of the 5mCAdpBS-Seq libraries were converted into bed files which were then intersected using bedtools intersect (version 2.30.0) (33) for 11 genomic elements: SINEs, LINEs, simple repeats, exons, introns, intergenic sequences, promoters, CpG islands, 5′ untranslated region (UTR), 3′ UTR, and transcription termination site (TTS). The intersection counts were used to calculate the percentage of CpG-containing fragments. Note that certain elements were potentially counted in duplicate due to overlap in regions (e.g. promoters and CpG islands).

### Different CpG methylation fragment overlap with genomic elements

Fragments were binned into four categories by their CpG methylation status based on the SAM flag status (0%, >0 to 25%, >25–75% and >75–100%). The different bins were then intersected using bedtools intersect (version 2.30.0) (33) with ‘-wo’ and ‘.bed’ for elements related to genes (CpG shelf, CpG shore, CpG island, promoter, 5′ UTR, first exon, all exons, introns, 3′ UTR, TTS, and intergenic regions).

### Epigenetic marks

Epigenetic marks were calculated using the bedtools intersect function with ‘-wo’ (version 2.30.0) (33). Intersected base counts were divided by the total base counts in the bed file. Control bed files were generated using bedtools shuffle with the human reference (GenBank: GCA_000001305.2, GRCh38) and by sourcing the fragment size counts and distribution from the uscfDNA or mncfDNA bed file for each sample. The observed ratio was calculated by dividing the percentage of intersecting bases in the bed file by the percentage of intersecting bases in randomly shuffled control bed files for each epigenetic mark in the uscfDNA and mncfDNA bins. Experiment reference files were retrieved from the BLUEPRINT Data Analysis Portal (35). Specific subjects were eosinophil (S006XE53 and S006XEH2), macrophage (C005VG51 and C005VGH1), monocyte (C000S5A1b and C000S5H2), and neutrophil (C0010KA1bs and C0010KH2). The percent of intersected base pairs was normalized against control shuffled bed files to compare non-cancer and NSCLC samples.

### Enrichment of different CpG methylation % bins over the TSS

The pattern of CpG fragment enrichment –1000 bases upstream and +1000 bases downstream from the TSS differ among uscfDNA and mncfDNA fragments and correlate with gene activity. Plots were generated using SeqMonk (version 1.48.1, https://www.bioinformatics.babraham.ac.uk/projects/seqmonk/). Bed files from different methylation bins (0%, >0–25%, >25–75% and >75–100%) were loaded, and probes were defined using ‘Feature Probe Generator’ and designed around bed files of all TSSs or different sets of TSSs in genes with different expression levels. Probes were designed over features from –1000 bp to +1000 bp, quantified for their enrichment, and plotted using ‘probe trend plot’ with ‘Force plot to be relative’ (Figure 5) or ‘scale within each data store’ (Figure 8). Sets of TSSs were categorized according to RNA-Seq data from genes in the PBMCs from the buffy coat as described elsewhere (36). High expression was considered to be >41.07 TPM, medium 15.36–41.06 TPM, low 1–15.36 TPM, and silent <0 TPM. The list of TSSs was taken from Homer hg38 bed files (37).

### Plotting CpG methylation % quantification trends

Plots were generated using SeqMonk (version 1.48.1, https://www.bioinformatics.babraham.ac.uk/projects/seqmonk/). The CGmap.gz files generated by BSBolt were converted to cg.bismark.cov.gz files. The files were imported, and SeqMonk in-silico probes were first defined using Feature Probe Generator for the different genomic elements of interest from –5000 bases to +5000 bases (Make probes ‘over features’ selected). The existing probes were then further defined using the ‘Running Window Generator’ with the following settings: probe size was set at 100 bp and step size was set at 100 bp, and limit by region set to ‘active probe list’. Afterwards, ‘features to quantitate’ was selected for the existing probes with the following settings: minimum count to include position was set to 1, minimum observations to include feature was set to 1, and combined value to report as mean. Next, quantification trend plots were constructed for the genomic elements. The option to remove exact duplicates was checked, and probes were made from –5000 bases upstream to +5000 bases downstream from the body of the feature.

### Differentially methylated regions

Samples were aggregated using metilene_input.pl from the metilene package (38) using a minimum coverage of 1. Differentially methylated regions (DMRs) between samples were identified using with the settings ‘-mincpgs 3’, ‘–maxdist 100’, ‘–minMethDiff 0.1’ and ‘–valley 0.7’. Closest genes were analyzed using bedtools closest with default settings and an hg38 gene reference from UCSC RefSeq (refgene; from https://genome.ucsc.edu/cgi-bin/hgTables).

### Deconvolution of tissue of origin

Samples were analyzed using the CelFiE (CELl Free DNA Estimation via expectation-maximization) algorithm with default parameters as described elsewhere (39).

### Statistical analysis

For genomic element profiles and observed ratios of epigenetic marks, Tukey's multiple comparison test was performed after two-way ANOVA. Individual Student *t*-tests were performed for the different tissue types for the percentage of predicted tissue deconvolution and CpG methylation of G-Quad-containing fragments. Error bars represent the SEM for an average of five non-cancer controls and four NSCLC samples.

### Data and code availability

The sequencing data were deposited in the National Institute of Health Sequence Read Archive under accession number PRJNA980280 and GEO accession number GSE252088. Processing scripts and analysis commands are found at: https://github.com/WlabUCLA/BRcfDNA-Seq.

## Results

### Merging paired-end reads prior to alignment impacts the fragment length profile of BS-treated cfDNA libraries

Depending on the length of the cfDNA fragment, paired-end sequencing cycles may only report a fraction of the DNA sequence (Supplementary Figure S1A), whereas certain circumstances lead to fragments being excluded from further processing (Supplementary Figure S1B). We compared a paired-end mapping pipeline to merged paired reads prior to alignment (Supplementary Figure S1C–F). For BS-Seq libraries, two distinct peaks were present (150 bases and 167 bases) in the mncfDNA region (Supplementary Figure S1D). However, a different pattern was observed with merged pre-alignment compared to the standard paired-end processing.

The BRcfDNA-Seq libraries had comparable MAPQ scores in the two analysis modes, with the scores for the merged-reads protocol being slightly lower (Supplementary Figures S1E and F). For BS-Seq, both default and merged processing had lower MAPQ scores for bins from 30 to 39 bases but stabilized for the bins >40 bases. The merged reads were slightly lower than with the paired-end processing.

The percentage of total reads for different workflows (BRcfDNA-Seq or BS-Seq) were compared along the bioinformatics pipeline (Supplementary Figures S2A and D). A proportion of reads (72.6% ± 3.2%) was kept after the initial merging step (Supplementary Figure S2B and E). Compared to unmerged analysis, merged processing universally resulted in fewer final usable reads (unmerged: 51.9 ± 4.7% versus merged: 46.6 ± 3.6%; Supplementary Figure S2C and F). However, subsequent steps after merging (quality control and alignment) retained more reads than the paired-end pipeline. This led us to process all sequenced samples with the merged pipeline to alleviate the artificial double peak generated in the mononucleosomal region (Supplementary Figure S1D).

### The 5mCAdpBS-seq protocol reduces the inclusion of degraded DNA in the uscfDNA region of the final library

**Figure 1.**
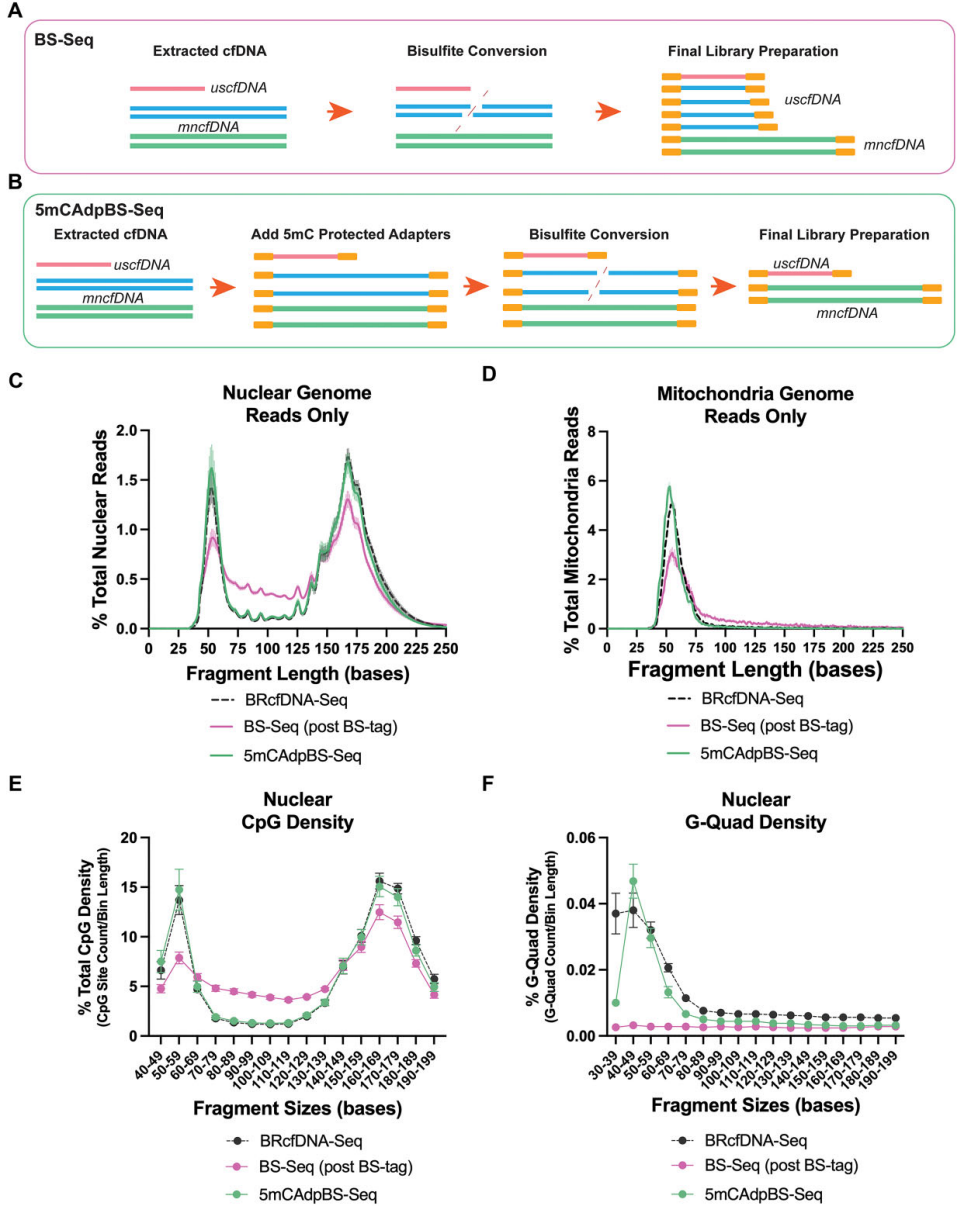
5mCAdpBS-Seq protocol reduces the inclusion of DNA degradation in the ultrashort region of the final library. (**A**) Schematic of the routine BS-Seq workflow incorporates degraded cell-free DNA or genomic DNA, which enters the library, potentially masking the uscfDNA methylation signal. (**B**) 5mCAdpBS-Seq protocol in which pre-methylated adapters are attached prior to bisulfite conversion, preventing degraded DNA from entering the final library. (**C**) BRcfDNA-Seq (black), BS-Seq (pink), and 5mCAdpBS-Seq (green) protocols generate different fragment profiles for reads aligning to nuclear and (**D**) mitochondria genomes. (**E**) CpG density and (**F**) G-Quad density of 5mCAdpBS-Seq resemble the BRcfDNA-Seq profiles compared to the BS-Seq protocol. The reads that align to mitochondria contributed to the minority of the total sequence reads, averaging 0.35 ± 0.006%, 0.0135 ± 0.002% and 0.0664 ± 0.012% for BRcfDNA-Seq, BS-Seq and 5mCAdpBS-Seq, respectively. Data are presented as the mean and SEM of five paired non-cancer samples.

As the initial BS-Seq experiments indicated differences in the size distribution compared to non-BS BRcfDNA-Seq (Supplementary Figure S1C and D), we hypothesized that the increased representation of fragments in the 70–130 base region derived from larger cfDNA degradation during the bisulfite treatment process (Figure 1A) (40). To this end, we tested whether ligating single-stranded 5mC-protected adapters prior to bisulfite treatment (5mCAdpBS-Seq) would reduce the incorporation of degraded DNA, which could be misclassified as uscfDNA (Figure 1B).

We assessed the *in silico* read loss between the BS-Seq and 5mCAdpBS-Seq protocols. Compared to BS-Seq, the 5mCAdpBS-Seq protocol showed a greater read loss in most processing steps (merging, quality control, and alignment) (67.8 ± 3.4% for BS-Seq versus 54.6 ± 5.3% for 5mCAdpBS-Seq reads remaining). However, after the removal of PCR-duplicated reads (de-duplication based on UMIs), the remaining reads between both protocols were comparable (46.6 ± 3.6% for BS-Seq versus 45 ± 4.7% for 5mCAdpBS-Seq reads remaining; Supplementary Figure S3A). In some cases, the percent remaining reads was higher for the 5mCAdpBS-Seq protocol than the BS-Seq protocol (Supplementary Figure S3B).

Reads obtained from the 5mCAdpBS-Seq protocol after aligning to nuclear DNA showed substantial differences in the fragment profile compared to BS-Seq while closely resembling the BRcfDNA-Seq protocol (Figure 1C). In particular, the region from 70 to 130 bases was largely absent from the DNA degradation seen in the BS-Seq protocol profiles (Figure 1C, Supplementary Figure S1D).

The percent of total genomic coverage at each 100k base bin along the genome was compared between the three protocols (Supplementary Figure S4A, B, D and E). For uscfDNA fragments, on average, the 5mCAdpBS-Seq protocol had a greater *R*^2^ coefficient compared to BS-Seq, demonstrating a closer similarity to BRcfDNA-Seq (Supplementary Figure S4C) but no trend for mncfDNA (Supplementary Figure S4F).

Alongside nuclear DNA, the mitochondrial genome (mitDNA) also contributes to the pool of cfDNA in circulation (41). However, the reads that align to mitDNA only represent a minor fraction of the total sequence reads, averaging 0.35 ± 0.006%, 0.0135 ± 0.002% and 0.0664 ± 0.012% for BRcfDNA-Seq, BS-Seq and 5mCAdpBS-Seq, respectively. The reads aligned to the mitDNA of 5mCAdpBS-Seq closely resembled the BRcfDNA-Seq fragment distribution pattern with a slight peak shift to the left (Figure 1D). In comparison, the BS-Seq mitDNA profile had a peak at 57 bases, with most fragments occupying between 40 and 75 bases. Compared to the nuclear uscfDNA, the mitDNA fragment curve was not symmetrical, with a more prominent shoulder at ∼60 bases.

### Genomic characteristics of the 5mCAdpBS-Seq protocol closely resemble BRcfDNA-Seq

We examined the pattern of CpG density at each fragment size bin and observed that the 5mCAdpBS-Seq protocol reads followed the same pattern as those for BRcfDNA-Seq (Figure 1E), whereas the BS-Seq protocol had a lower peak at 50 to 59 bases but an elevated CpG density from 70 to 130 bases. This pattern resembled that of the fragment size distribution, in line with the hypothesis that bisulfite treatment leads to fragmented genomic and mncfDNA, contributing to the uscfDNA region (Figure 1C).

Previous reports indicate that the uscfDNA is enriched in G-rich sequences that can potentially form G-Quad secondary structures (13). G-Quad signatures were enriched in our BRcfDNA-Seq and 5mCAdpBS-Seq protocols, with the highest peak in the bin comprising 40–49 bases (Figure 1F). However, in the same samples processed by BS-Seq, this enrichment was absent in the ultrashort region.

### BRcfDNA-seq and 5mCAdpBS-seq protocols show that uscfDNA maps to regions associated with active genes compared to mncfDNA

**Figure 2.**
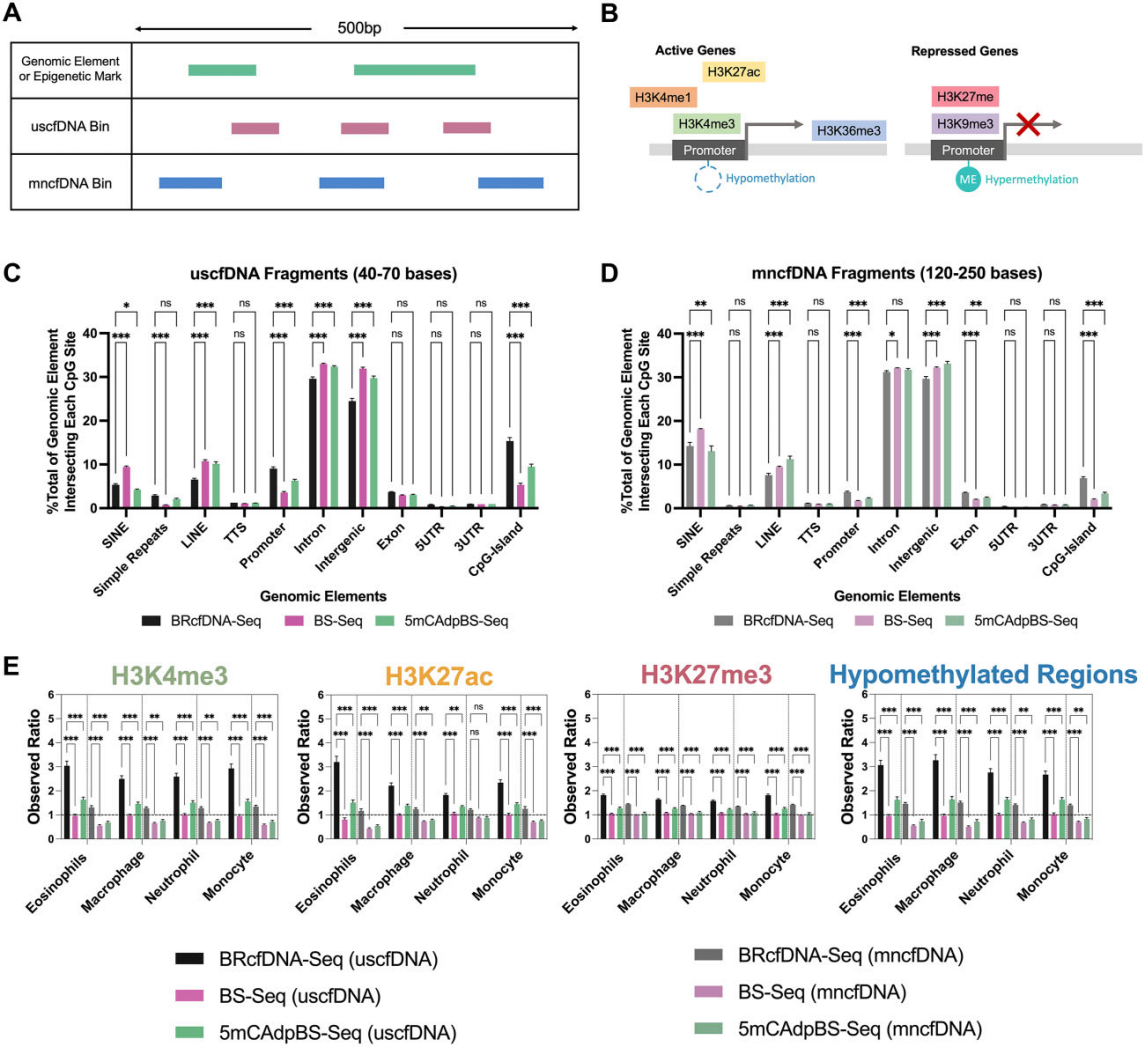
The 5mCAdpBS-Seq protocol resembles BRcfDNA-Seq in regard to the coverage of genomic elements and epigenetic marks. (**A**) Schematic of intersection methodology to determine where uscfDNA or mncfDNA bases overlap with genomic elements and epigenetic mark regions in reference bed files from genomic or ChIP-seq databases. (**B**) Genomic regions derived from epigenetic marks, including methylation patterns and histone modifications, are associated with active or repressed gene activity. (**C**) The percent composition of genomic elements at each CpG-site was compared between BRcfDNA-Seq (black), BS-Seq (pink), and 5mCAdpBS-Seq (green) protocols for uscfDNA bins (40–70 bases) and (**D**) mncfDNA bins (120–250 bases). SINE: short interspersed nuclear element, LINE: long interspersed nuclear element, TTS: transcription termination site, 5′UTR: 5′ untranslated region, 3′UTR: 3′ untranslated region. (**E**) Observed ratio (% of intersecting bases in bed file to % intersecting bases in randomly shuffled control bed files) for each epigenetic mark in uscfDNA and mncfDNA bins. Randomly shuffled bed files were generated for each sample to act as a control for intersection locations. The horizontal dotted line represents the observed ratio of 1.0. Data are presented as the mean and SEM of five paired non-cancer samples. * *P*< 0.05, ** *P*< 0.01, *** *P*< 0.001, Tukey's multiple comparison test after two-way ANOVA. Only comparisons with BRcfDNA-Seq are shown.

We compared the intersection profiles of CpG sites and cfDNA fragments (Figure 2A) with genomic elements and epigenetic marks (Figure 2B) for all three protocols for both the uscfDNA and mncfDNA populations (Figure 2C and D). For both cfDNA populations, the 5mCAdpBS-Seq protocol more accurately recapitulated the genomic element profile compared to BS-Seq for SINEs, promoters, exons and CpG islands. In addition, uscfDNA fragments appeared to be significantly enriched in promoters, exons, simple repeats, and CpG islands, whereas mncfDNA was enriched more in SINEs and intergenic regions.

As uscfDNA appears to be enriched in promoters (Figure 2C) (11,13–15), we further examined whether the results obtained with 5mCAdpBS-Seq protocol resembled those of BRcfDNA-Seq in genomic regions associated with increased gene activity (Figure 2B) (42). We observed that the mapping patterns for epigenetic marks with the 5mCAdpBS-Seq protocol more closely reflected the patterns obtained using BRcfDNA-Seq in comparison to the mapping patterns of BS-Seq with BRcfDNA-Seq (Figure 2E and Supplementary Figure S5) for both uscfDNA and mncfDNA fragments. The uscfDNA demonstrated a higher intersection percentage versus a matched shuffled position control with active gene epigenetic marks (H3K4me1, H3K4me3, H3K27ac modifications and hypomethylated regions), whereas mncfDNA had the opposite trend. In contrast, for repressed gene epigenetic marks, both uscfDNA and mncfDNA showed an increased intersection percentage with H3K27me3, H3K9me3, and hypermethylated regions.

Based on these findings of apparent similarity between 5mCAdpBS-Seq and BRcfDNA-Seq, all subsequent analyses were performed on the data obtained using the pre-methylated adapter protocol.

### Nuclear uscfDNA fragments are globally hypomethylated in the 5mCAdpBS-Seq protocol

**Figure 3.**
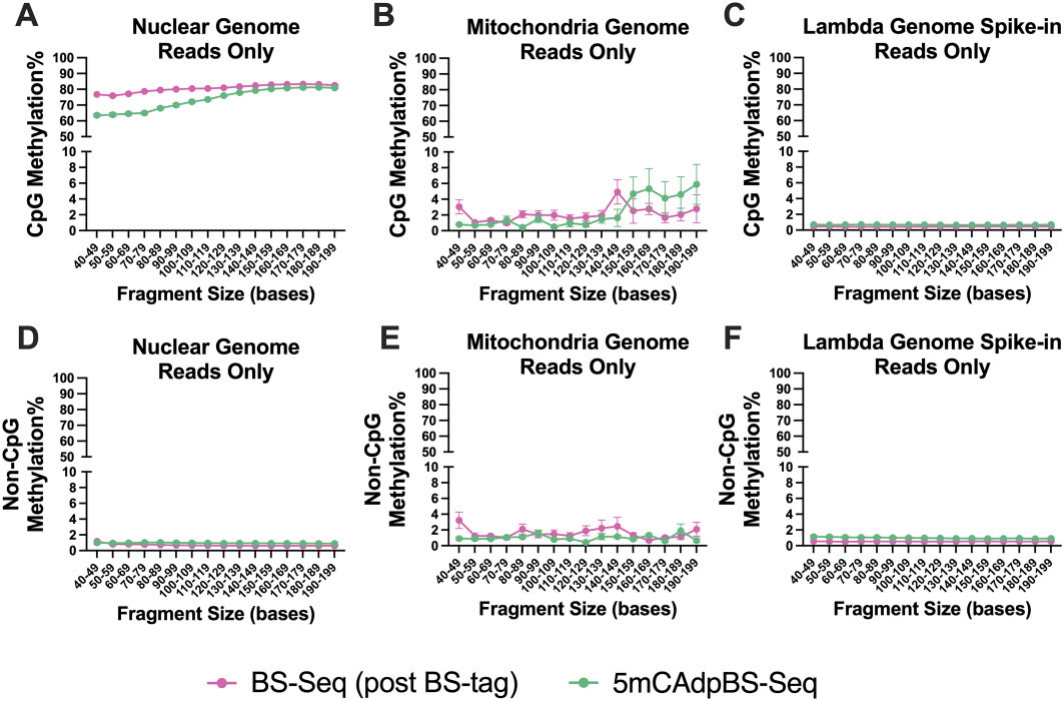
Compared to the BS-Seq protocol, the 5mCAdpBS-Seq protocol shows global hypomethylation of nuclear uscfDNA fragments. (**A–C**) CpG methylation and (**D–F**) non-CpG methylation for nuclear, mitochondrial, and lambda genome spike-in using BS-Seq and 5mCAdpBS-Seq protocols in 10-base increment bins from 40–200. Lambda spike-in control indicates the inherent noise of bisulfite conversion. Data are from five paired samples undergoing both protocols. Error bars indicate SEM for five samples.

UscfDNA fragments had lower mean CpG methylation% in the 5mCAdpBS-Seq profiles than in the BS-Seq protocol (63.6–64.6% versus 76.8–77.1%; Figure 3A). In contrast, mncfDNA fragments (120–200 bases) had a similar CpG methylation% between both protocols (80.2–80.9% versus 80.5–82.5%) and were higher than in the uscfDNA population. The nuclear non-CpG methylation in both protocols was approximately 1% (Figure 3D and Supplementary Figure S6A).

Reads aligning to the mitDNA (Figure 3B and Supplementary Figure S6B) were used as a biological control because the mitDNA is expected to be hypomethylated (43,44). For both protocols, the CpG and non-CpG methylation levels were < 5%, with fluctuations for bins > 130 bases (Figures 3B and E and Supplementary Figure S6B and C). However, there were few reads beyond 130 bases.

As a negative control, enzymatically sheared lambda phage DNA was spiked into plasma samples undergoing the BS-Seq or 5mCAdpBS-Seq protocol to determine the bisulfite conversion efficiency (Figure 3C and F and Supplementary Figure S6D and E). The mean CpG methylation% was similar for both protocols, <1% for CpG and < 1.5% for non-CpG methylation%, though it appeared slightly higher for 5mCAdpBS-Seq.

### CpG methylation levels in uscfDNA are lower than in mncfDNA, with differing patterns for genomic elements

Using the 5mCAdpBS-Seq protocol, we constructed karyograms showing differences in the percent coverage for CpG sites in uscfDNA and mncfDNA (Supplementary Figure S7A and B). Of the uscfDNA CpG site positions, 41.4 ± 5% of uscfDNA sites could be found within the mncfDNA population, but most sites were unique to mncfDNA (Supplementary Figure S7C).

**Figure 4.**
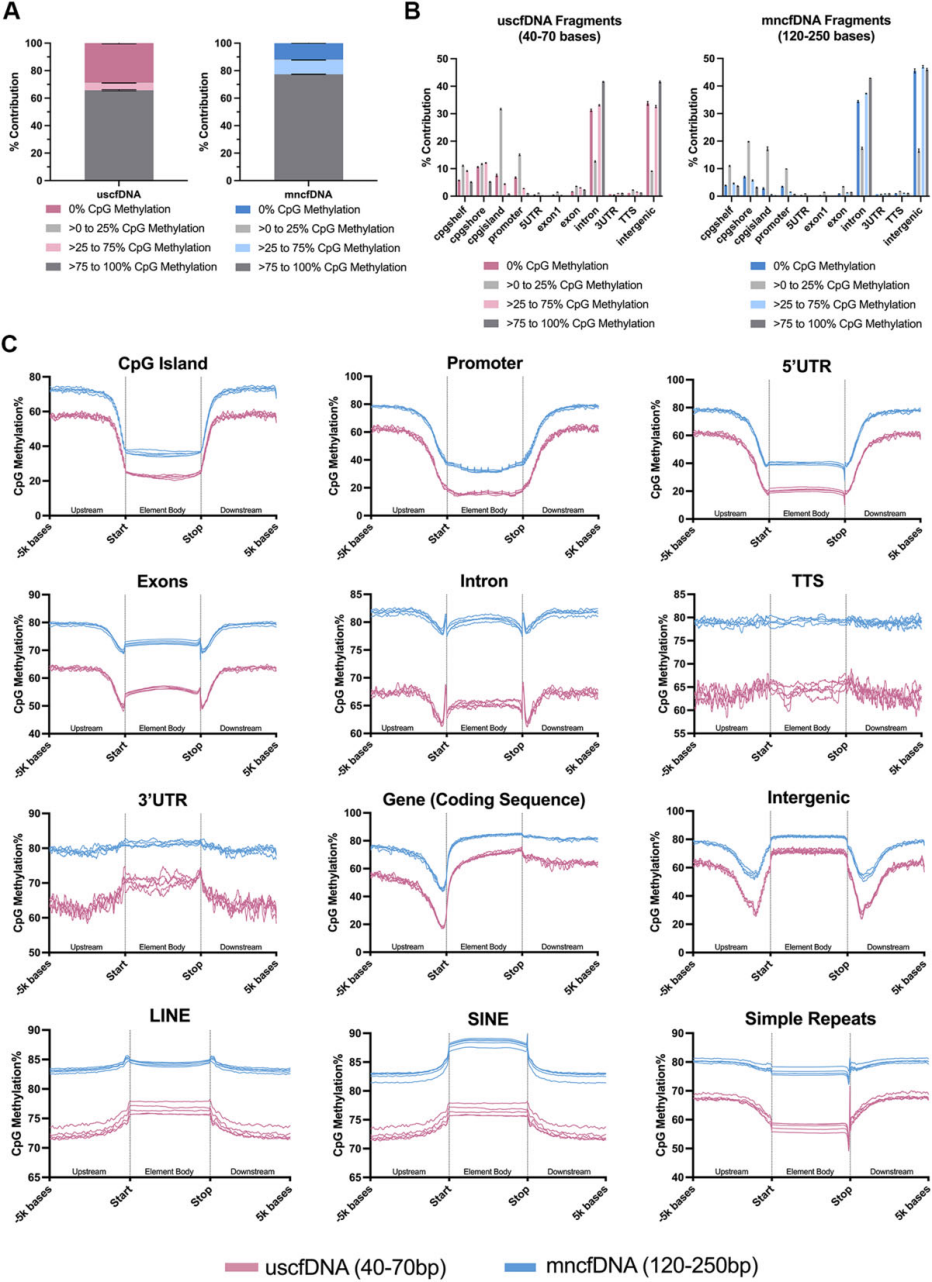
CpG methylation patterns differ between uscfDNA and mncfDNA fragments. (**A**) Differences in the percent composition of different methylated CpG read categories between uscfDNA and mncfDNA fragments. (**B**) Percent composition of select genomic elements of different methylated read categories along select elements of the typical gene structure. (**C**) The average CpG methylation% patterns from 5000 bases upstream and 5000 bases downstream from the body of the element for uscfDNA and mncfDNA-sized reads. Lines show five separate non-cancer samples processed with the 5mCAdpBS-Seq protocol.

Both uscfDNA and mncfDNA fragments were subdivided into four CpG methylation categories (0%, >0–25%, >25–75% and >75–100%; Figure 4A). Recapitulating the global CpG methylation, uscfDNA fragments had a greater proportion of 0% methylation and a subsequently lower proportion of >75 to 100% CpG methylation fragments compared to mncfDNA. When intersected against gene regulatory elements, a larger proportion of uscfDNA fragments of all methylation statuses converged around CpG elements and promoters (Figure 4B). Interestingly, despite contributing <0.5% of total fragments, the >0–25% CpG methylation fragments were enriched in CpG elements (island, shore or shelf).

The behaviors of various genomic elements of interest were examined by plotting the CpG methylation% from 5000 bases upstream from the start of the body of element to 5000 bases downstream from the end of body of the element (Figure 4C). In general, the CpG methylation% of uscfDNA fragments was 10–20% lower than for mncfDNA over the same regions, mirroring the genome-wide CpG methylation state (Figure 3A). The general patterns of the CpG methylation distribution were similar, though uscfDNA had more variance, most likely due to reduced coverage. The three most distinct methylation patterns between the two cfDNA populations were simple repeats, LINEs, intergenic regions and exons.

### The uscfDNA fragments are enriched directly upstream of the TSS and reflect gene expression activity

**Figure 5.**
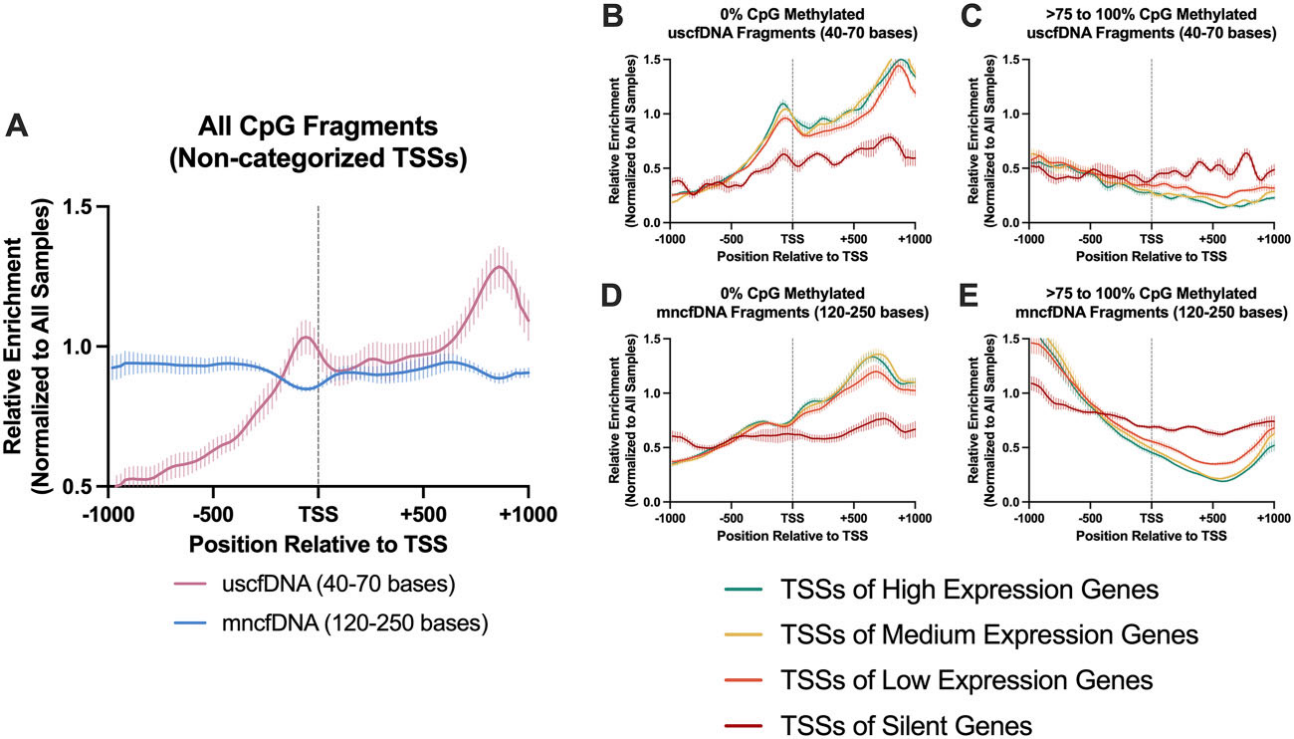
The patterns of CpG fragment enrichment -1000 bases upstream and + 1000 bases downstream from the transcription start site (TSS) differ among uscfDNA and mncfDNA fragments and correlate with gene activity. (**A**) uscfDNA fragments are enriched upstream from the average TSS compared to mncfDNA fragments. (**B**) The enrichment of 0% methylated uscfDNA and (**D**) mncfDNA fragments positively correlates with the TSSs of high expression genes, whereas the >75–100% CpG-methylated fragments are negatively correlated in the (**C**) uscfDNA and (**E**) mncfDNA fragments based on the RNA expression activity from RNA-Seq experiments on the buffy coat in previous literature. High expression >41.07 TPM, medium 15.36–41.06 TPM, low 1–15.36 TPM, and silent <0 TPM. Lines show five separate non-cancer samples processed by the 5mCAdpBS-Seq protocol. Enrichment was normalized to all samples in the comparison.

Unlike mncfDNA fragments which demonstrate decreased coverage over TSSs, uscfDNA fragments exhibit enrichment (13,45). When examining only CpG-containing fragments, we observed a similar pattern for uscfDNA fragments, showing an upward inflection (Figure 5A). The pattern of coverage of uscfDNA and mncfDNA fragments binned by 0% or >75–100% CpG methylation were correlated with TSSs grouped by varying gene expression levels (from hemopoietic cells) (Figure 5B–E). We observed that the enrichment of 0% CpG-methylated uscfDNA near the TSS positively correlated with highly expressed genes (Figure 5B), and the >75–100% CpG-methylated fragments were negatively correlated. In contrast, the 0% CpG-methylated mncfDNA showed more pronounced depression towards the TSSs of genes with high expression (Figure 5D). In general, the profile of fragment enrichment for TSSs in genes with low expression was more horizontally stable along the TSS regardless of the CpG methylation state (Figure 5B–E). These patterns were less pronounced in the >0–25% and >25–75% methylated fragments (Supplementary Figure S8).

### Differentially methylated regions exist between uscfDNA and mncfDNA

**Figure 6.**
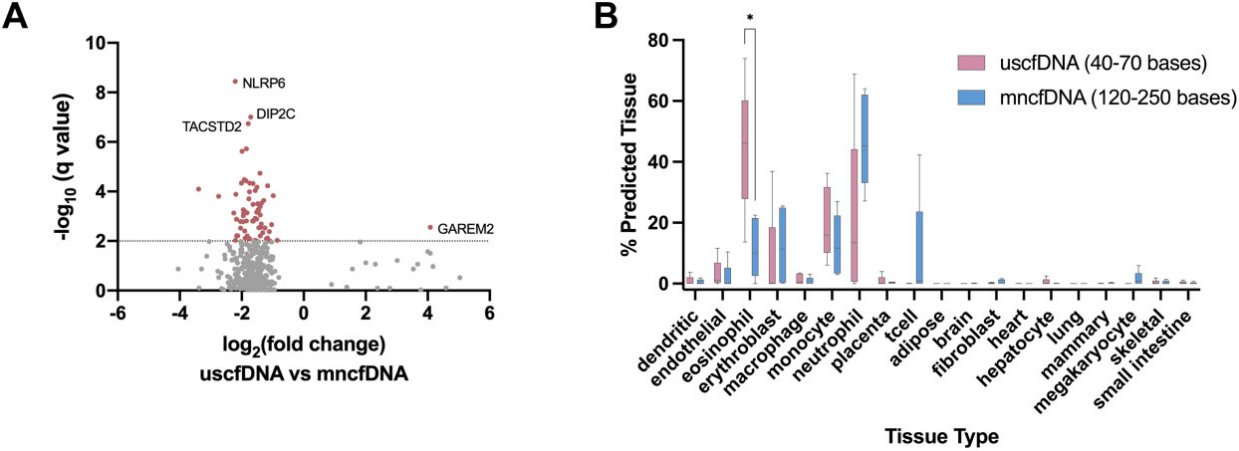
Differentially methylated regions show differences in genes and cell-of-origin. (**A**) Differentially methylated region analysis between merged uscfDNA and mncfDNA bam files from five samples, showing 68 significant DMRs (*q*-value < 0.01) and their closest gene. Only candidates with *q*-value <1.0 are shown. (**B**) Box and whisker plots of CelFiE deconvolution prediction of the blood cell tissue-of-origin signal from the methylation patterns in uscfDNA and mncfDNA. The prediction reveals that uscfDNA and mncfDNA are derived from blood cell DNA. Non-paired multiple paired *t*-tests were used to compare the percent contribution of cell type between uscfDNA and mncfDNA. * *P*< 0.05 (unadjusted). Errors bars indicate the min and max from five non-cancer samples that underwent the 5mCAdpBS-Seq protocol.

As a minor overlap existed between uscfDNA and mncfDNA CpG sites, we aggregated the bam files for the uscfDNA and mncfDNA from five samples and analyzed regions that were differentially methylated (DMRs) between the two cfDNA populations (Figure 6A). Sixty-eight significant DMRs were found, the majority of which were hypomethylated in uscfDNA compared to mncfDNA. Moreover, the majority of DMRs were in close proximity to TSSs (Supplementary Figure S9).

### Deconvolution suggests that uscfDNA is mainly derived from peripheral blood cells

Next, we attempted to deconvolute the fragments from the uscfDNA and mncfDNA populations into their cell/tissue-of-origin using the CpG methylation patterns (Figure 6B). The CelFiE algorithm (39) was constructed as an expectation-maximization algorithm, which iteratively finds the vector of tissue proportions with the greatest maximum likelihood using information from both the reference data supplied at the time of running the algorithm and the regions of the genome that are variable between tissues using whole genome bisulfite sequencing data, for different tissues, obtained from ENCODE and Blueprint (35,46). Using this algorithm, which was designed to deconvolute signals from low input cfDNA samples, we confirmed that the major tissue-of-origin for both uscfDNA and mncfDNA is hematopoeitic. This methylation tissue-of-origin analysis indicated contributions of uscfDNA from eosinophils, erythroblasts, monocytes, neutrophils and T cells. Moreover, CelFiE indicated that uscfDNA has significantly more fragments originating from eosinophils compared with mncfDNA.

### uscfDNA CpG mapping patterns and methylation characteristics can discriminate non-cancer samples from late-stage NSCLC

As a proof of concept, we examined whether the methylation profile of uscfDNA would be an effective biomarker for cancer detection. We processed four late-stage NSCLC samples using the 5mCAdpBS-Seq protocol and compared them with non-cancer samples. The global fragment patterns showed an elevated uscfDNA peak in the NSCLC samples and a lower rightward shoulder in the mncfDNA regions of 175–200 bases (Supplementary Figure S10A). For reads mapping to the nuclear genome, in the 10-base bins between 40 bases and 140 bases, the NSCLC samples appeared to have higher percentage of CpG methylation (4–6%) compared to the non-cancer samples at sizes <140 bases (Supplementary Figure S10B).

**Figure 7.**
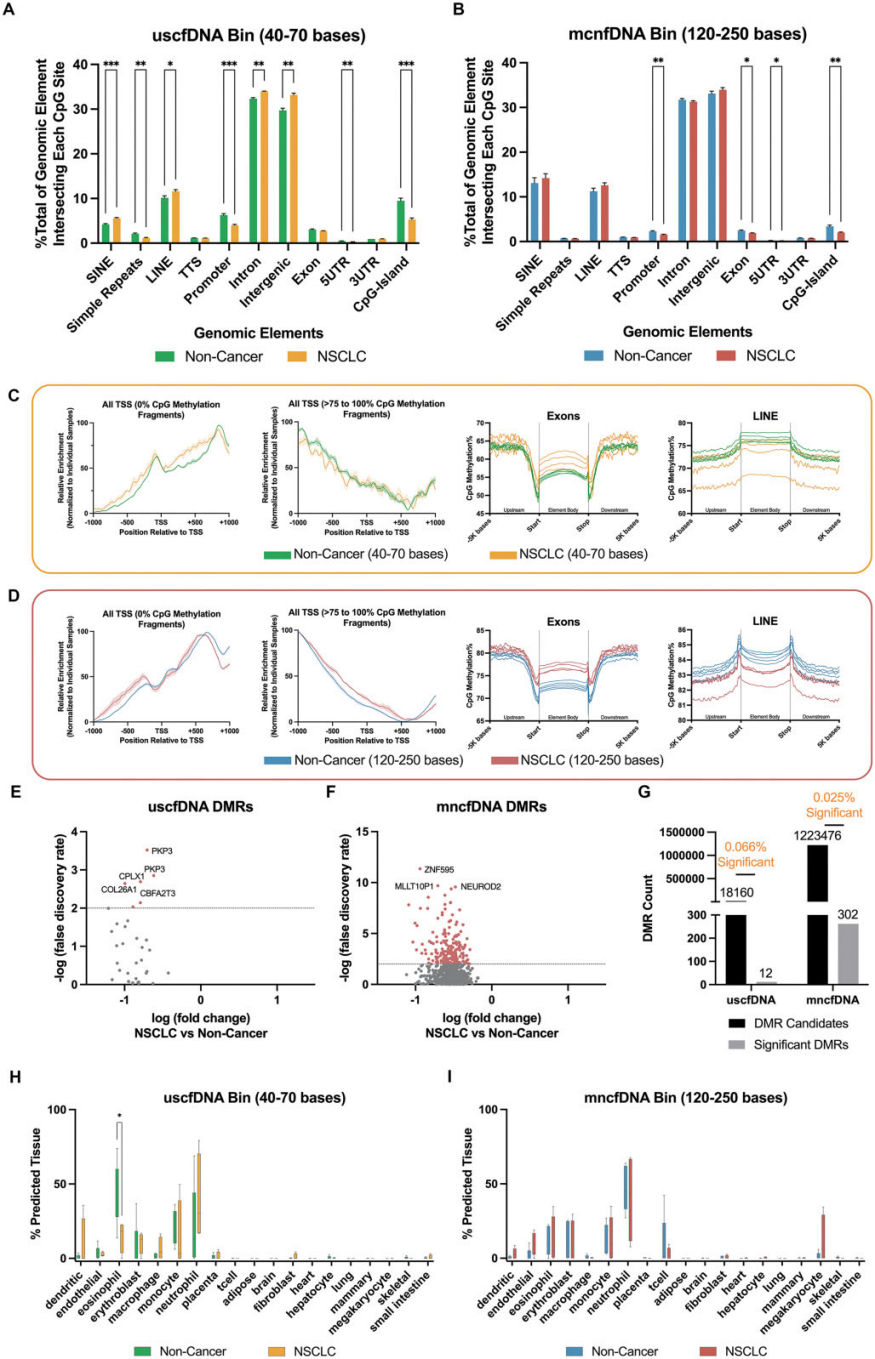
CpG coverage and methylation patterns differ between non-cancer and NSCLC samples. (**A**) The composition of different genomic element category locations where CpG site-containing read alignments were compared between the NSCLC and non-cancer samples for uscfDNA and (**B**) mncfDNA. (**C**) The pattern of enrichment of CpG fragments –1000 bases upstream and +1000 bases downstream and average CpG methylation% patterns from 5000 bases upstream and 5000 bases downstream of the body of the exon and LINEs differed among NSCLC and non-cancer samples for uscfDNA and (**D**) mncfDNA fragments. (**E**) Differentially methylated region analysis between merged uscfDNA and mncfDNA bam files from five non-cancer and four NSCLC samples revealed significant DMRs in the uscfDNA and (**F**) mncfDNA bins (*q*-value < 0.01, only candidates with *q*-value < 1.0 are shown). (**G**) uscfDNA has a higher proportion of significant DMRs than mncfDNA. (**H**) Box and whisker plot of the CelFiE deconvolution algorithm suggests changes in cell type composition between non-cancer and NSCLC samples for the uscfDNA and (**I**) mncfDNA-sized bins. * *P*< 0.05, ** *P*< 0.01, *** *P*< 0.001, Tukey's multiple comparison test after two-way ANOVA (A and B). For the CelFiE deconvolution, error bars represent min and max positions with individual samples and unadjusted non-paired Student *t*-tests. Data are presented as the mean and SEM of five paired non-cancer and four NSCLC plasma samples. * *P*< 0.05, ** *P*< 0.01, *** *P*< 0.001 (unadjusted).

We identified eight types of genomic regions that were differentially represented by uscfDNA (Figure 7A). In contrast, only four regions showed differential representation in mncfDNA (Figure 7B). In the uscfDNA bin, there were significant changes in the proportion of SINEs, simple repeats, LINEs, promoters, introns, intergenic regions, 5′UTR, and CpG islands. In the mncfDNA bin, promoters, exons, 5′UTR and CpG island proportions appeared to be significantly different between NSCLC and non-cancer cohorts.

### Methylation patterns of genomic elements are altered in NSCLC

When we examined the enrichment around TSS and CpG methylation% patterns for uscfDNA (Figure 7C and Supplementary Figure S11A) and mncfDNA bins (Figure 7D and Supplementary Figure S11B Figure), the TSS was altered in the NSCLC samples for both the 0% and >75–100% CpG-methylated fragments. The NSCLC samples showed greater methylation variability, whereas the non-cancer samples were more uniform. For promoters, 5′UTR, exons and LINEs, hypermethylation was observed towards the body of the element in NSCLC samples compared to the non-cancer samples, but this observation was more evident in the mncfDNA bins. For the LINEs, the NSCLC samples appeared more hypomethylated compared to the non-cancer samples, but this was more apparent in the mncfDNA bin (Supplementary Figure S11A). In contrast, the flanking regions of SINEs were hypomethylated in NSCLC samples, which was more prominent in the uscfDNA bin than the mncfDNA bin. Introns, 3′UTR, and TTS elements were globally more hypermethylated in mncfDNA. In the uscfDNA, the methylation profile of the non-cancer samples was more uniform than the highly variable NSCLC traces.

### Differentially methylated regions and deconvolution are potential biomarkers for NSCLC detection

DMR analysis of the CpG methylation% of NSCLC and non-cancer samples revealed that both the uscfDNA and mncfDNA fragments had candidates for significant DMRs (Figure 7E and F). UscfDNA fragments had 12 significant DMRs out of 18 160 tested regions (0.066% significant) compared to mncfDNA, which had 302 significant DMRs out of 1223476 tested regions (0.025% significant) (Figure 7G). For both uscfDNA and mncfDNA, significant DMRs had lower methylation in NSCLC samples compared to non-cancer samples (Figure 7E and F). Some examples of the nearest candidate DMR genes were plakophilin3 (*PKP3*), complexin 1 (*CPLX1*), and collagen type XXVI alpha 1 chain (*COL26A1*). For mncfDNA, the top candidates based on q-value were zinc finger protein 595 (*ZNF595*), myeloid/lymphoid or mixed-lineage leukemia translocated to pseudogene 1 (*MLLT10P1*), and neuronal differentiation 2 (*NEUROD2*).

The CelFiE deconvolution prediction algorithm suggested differences in the tissue-of-origin profiles between the two cohorts (Figure 7H and I). In the uscfDNA fragment bin, the eosinophil signal appeared significantly decreased in NSCLC samples, matching the level found in the mncfDNA fraction. In contrast, in mncfDNA, there was a non-significantly increased megakaryocyte signal in some NSCLC samples.

### G-Quad-containing uscfDNA fragments show an increased CpG methylation% in NSCLC samples

**Figure 8.**
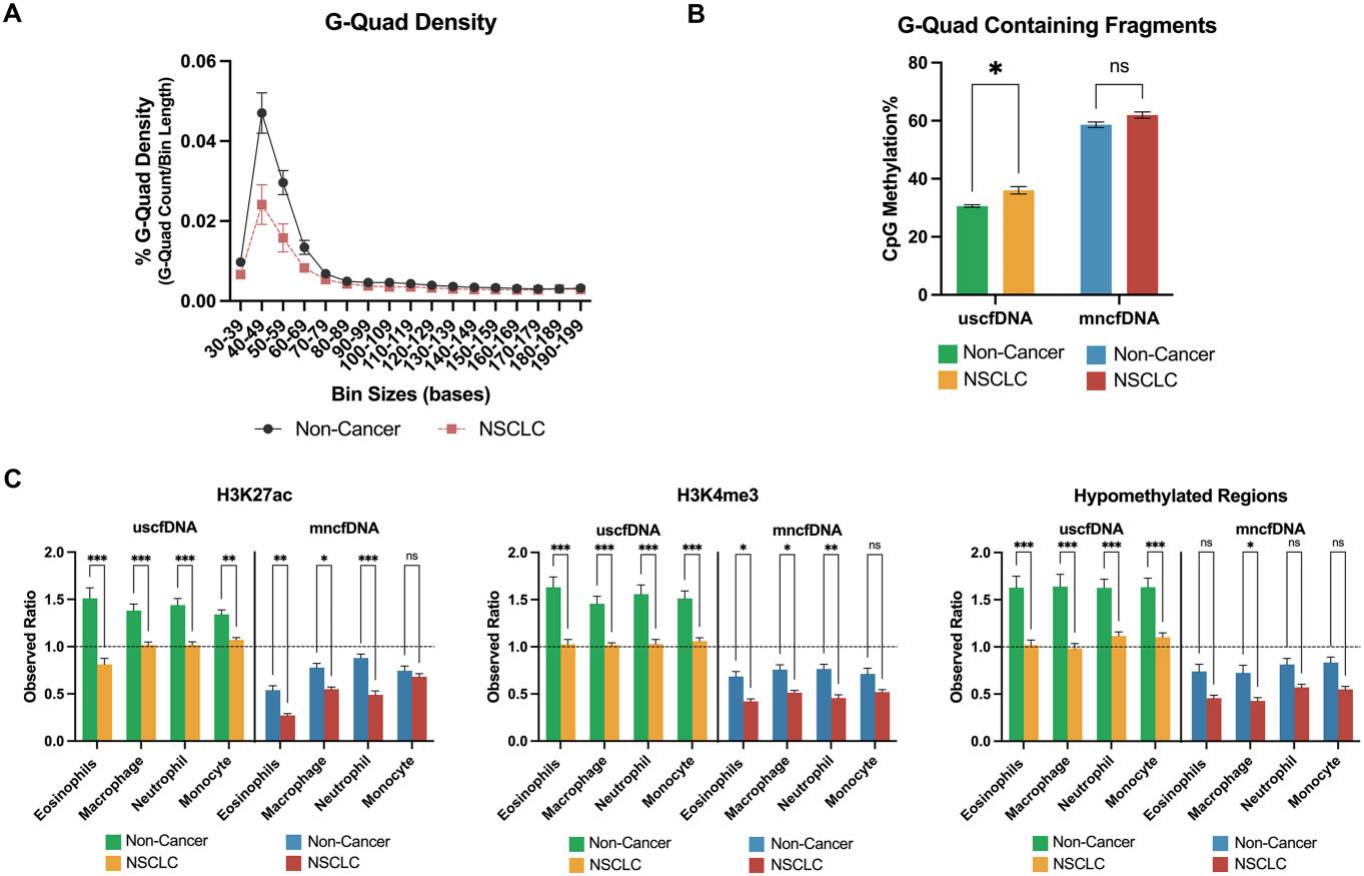
G-Quad methylation% and epigenetic mark overlap% are potential NSCLC biomarkers. (**A**) G-Quad density was decreased in the uscfDNA regions (40–70 bases) in NSCLC. (**B**) CpG methylation% significantly increased in G-Quad-containing fragments in uscfDNA. (**C**) Normalized percent of intersecting bases for three epigenetic marks (H3K27ac, H3K4me3 and hypomethylated regions) decreased in NSCLC samples in uscfDNA and mncfDNA bins. Observed ratio (% of intersecting bases in bed file to % intersecting bases in randomly shuffled control bed files) for each epigenetic mark for uscfDNA and mncfDNA bins. The horizontal dotted line represents the observed ratio of 1.0. Data are presented as the mean and SEM of five paired non-cancer and four NSCLC plasma samples. * *P*< 0.05, ** *P*< 0.01, *** *P*< 0.001, Student *t*-test (**B**) and Tukey's multiple comparison test after two-way ANOVA (**C**).

NSCLC samples had a smaller G-Quad signature in the uscfDNA region compared to non-cancer samples (Figure 8A). When the G-Quad-containing fragments were filtered out and analyzed for CpG methylation%, NSCLC samples were significantly hypermethylated in the uscfDNA fraction compared to non-cancer samples, whereas the difference was not significant in the mncfDNA fraction (Figure 8B).

### cfDNA overlap with cell type-specific epigenetic marks is altered in NSCLC

We also examined whether the normalized percent of intersecting base pairs for epigenetic marks was altered in NSCLC. Three epigenetic marks (H3K27ac, H3K4me3 and hypomethylated regions) had significantly decreased overlap in the uscfDNA and mncfDNA fractions in NSCLC samples (Figure 8C). A decrease in the percentage of intersection was also observed in H3K27me3, H3K36me, and H3K4me1 epigenetic marks in uscfDNA and mncfDNA, but to a lesser extent (Supplementary Figure S12). There did not appear to be any differences in the percentage of intersection of H3K9me3 or hypermethylated regions in NSCLC samples (Supplementary Figure S12).

## Discussion

Here, we described an optimized library preparation protocol for cfDNA in which single-stranded 5mC pre-methylated adapters were ligated to heat-denatured DNA fragments prior to bisulfite conversion and sequencing (i.e. 5mCAdpBS-Seq). This method improves the accuracy of downstream analysis by preventing degraded DNA from being incorporated into the final library and masking the methylation signal of uscfDNA. Using the 5mCAdpBS-Seq protocol, we observed that the CpG methylation% of uscfDNA is approximately 60%, compared to 70–80% for mncfDNA (Figure 3A). The unique methylation patterns, genomic location, and strandedness (11,12) suggest that uscfDNA originates through a different mechanism than mncfDNA, which is worth exploring further. The lower levels of DNA methylation observed in uscfDNA could be a result of the inherently lower methylation levels in genomic DNA due to expression activity, or further enzymatic modification, such as ten-eleven translocation (TET)-mediated DNA demethylation, after being ‘detached’ from genomic DNA or when entering circulation(47).

Methodologically, several strategies for directly detecting 5mC in cfDNA are limited to low throughput single-molecule techniques, such as nanopore sequencing (48) or single-molecule polymerase fluorescent labeling (49). Most methylation workflows require pre-treatment of the DNA fragments to indicate the CpG site methylation status. The main methods of pre-treatment are bisulfite treatment, methylation-sensitive restriction enzyme (MRE) digestion prior to bisulfite treatment (e.g. RRBS, MRE-BS), affinity enrichment, or other combinatory methods (Table 2). Targeted sequencing coupled with bisulfite conversion and microarray-based methods were not explored in this study because we wanted to initially examine the genome-wide profile of uscfDNA.

**Table 2.** Methylation analysis techniques for cfDNA

Technique	Single nucleotide resolution	Portrays non-CpG sites	DNA degradation	Optimized for uscfDNA
BS-Seq (used in this paper)	Yes	Yes	Yes	Not currently
5mCAdpBS-Seq (developed in this paper)	Yes	Yes	Yes	Yes
Reduced representation bisulfite sequencing (RRBS)	Yes	Yes*	Yes	Not currently
Circulating free methylated DNA immunoprecipitation sequencing (cfMeDIP-Seq)	No	No	No	Not currently
Methyl-CpG binding domain protein capture sequencing (MBD-Seq)	No	No	No	Not currently
Enzymatic methyl sequencing (EM-Seq)	Yes	Yes	No	Not currently
Targeted sequencing BS	Yes	Yes*	Yes	Not currently
Microarray-based	Yes	No	Yes	Not currently

*Only in enriched regions.

RRBS is based on the digestion of genomic DNA by methylation-insensitive restriction enzymes (e.g. MspI) with the intent to enrich CpG-dense regions (50). RRBS has been adapted to cfDNA analysis (18). However, RRBS is based on double-stranded DNA-cutting enzymes, so it is not compatible with uscfDNA methylation profiling unless a prior second-strand synthesis is incorporated. Other enzyme-based approaches, such as MRE-seq and MRE-BS-seq, suffer from the same problems as RRBS. Another strategy is to use 5mC-specific antibodies (meDIP-Seq) or methyl-binding proteins (MBD-Seq) to enrich the content of methylated DNA. In cfmeDIP-Seq, a monoclonal antibody against 5mC is immunoprecipitated with heat-denatured DNA and assessed by PCR, sequencing or an array (51). Alternatively, MethylCap uses GST-MBD fusion protein to capture methylated CpG-containing molecules (52). However, these techniques do not have single nucleotide resolution, and the small size of uscfDNA fragments can affect the immunoprecipitation efficiency (fewer CpG sites/fragment).

Enzymatic conversion promises lower DNA degradation and improved library yield but is time-consuming and may not have equivalent conversion efficiency (53). Our preliminary experiments with the enzymatic conversion did not generate libraries of sufficient quality to sequence (Supplementary Figure S13). Enzymatic conversion may not be optimized for single-stranded DNA (54) because the initial TET2 oxidation step has a preference for double-stranded DNA compared to single-stranded DNA or RNA (55). TET-assisted pyridine borate sequencing (TAPS) is another method that manipulates the identity of methylated CpG sites. 5mC and 5hmC are oxidized to 5-carboxylcytosine (5caC) and, using pyridine, borane is reduced to dihydrouracil (DHU). During a final PCR step, DHU is converted to thymine. To evaluate the applicability to uscfDNA, these conversion methods may need further optimization (56). For these reasons, we chose to proceed with a bisulfite-based methodology for the first foray into studying the methylation profile of uscfDNA.

Bioinformatically, the two-peaked mononucleosomal profile seen from the paired-end processing (Supplementary Figure S1D) could be explained by orphan reads of various lengths leading to a disproportioned accumulation of fragments up to a maximum of 150 bases. This pattern, up to the 150-base demarcation, matches the size distribution pattern of the merged protocol (Supplementary Figure S1C and D). Beyond 150 bases, the pattern also matches, but with a decreased abundance, suggesting an artifactual proportional decrease. We demonstrate that, if we filter for only properly paired reads, the size distribution pattern resembles the merged pipeline (Supplementary Figure S14). Therefore, the merged-read protocol not only ‘fixes’ the double-peak by removing these peaks, but also selects for fragments of high confidence because both paired reads must match to proceed with alignment.

When spiking with unmethylated non-human lambda DNA, the conversion efficiency was >99% and >98.5% for CpG and non-CpG methylation (Figure 3C and F). Interestingly, there was a slight increase in cytosine methylation levels in the 5mCAdpBS-Seq protocol for the digested lambda reads (but <0.8%) (Figure 3C and Supplemental Figure 6D). All experiments should use CpG methylation of lambda as a quality control for bisulfite conversion efficiency.

As the mitDNA has been described as containing low if any CpG methylation% (43,44), it can act as a biological internal negative control for the 5mCAdpBS-Seq protocol. We observed low levels (<2%) of both CpG and non-CpG methylation in mitochondrial cfDNA fragments (30–75 bases), suggesting that our workflow did not artificially over-represent methylation levels. There was a pattern of increasing methylation variability in fragments in bins >150 bases, potentially due to the lower number of reads in this fraction (Figure 3B, E and Supplementary Figure S4B and C).

Regarding bisulfite-induced degradation, higher molecular weight cfDNA has been documented to be more susceptible than mncfDNA (22). Therefore, during the BS-Seq protocol, the degraded DNA likely originated from these larger fragments of cfDNA. The CpG residues of genomic DNA are reportedly 70–80% hypermethylated (57), and both sources of degraded fragments (genomic DNA or high molecular weight DNA, which also derive from apoptosis) would still be expected to have these characteristics. This would explain why, during the BS-Seq protocol, the ‘bleeding’ of the degraded DNA into the 40–100 bases fraction skewed the average CpG methylation% higher towards 80%. In contrast, except for neurons and stem cells, non-CpG methylation is considered to be indistinguishable from non-conversion rates for most cell types, reflecting the low non-CpG methylation observed in this study (58) (Figure 3D and Supplementary Figure S6A).

Both the BS-Seq and 5mCAdpBS-Seq protocols indicated that uscfDNA has a lower CpG methylation%. Whether the lower CpG methylation% of uscfDNA is from an alternative mechanism separate from mncfDNA undergoing further fragmentation or uscfDNA is disproportionally derived from genomic regions tend to exhibit hypomethylated states during cell activity is unclear. Supporting the latter hypothesis, the uscfDNA fragments had enriched occupancy by regions categorized which can be hypomethylated such as simple repeats, promoters, exons, 5′UTRs and CpG islands, whereas the mncfDNA bin was enriched in potentially hypermethylated SINEs and intergenic elements (59). The enrichment in promoters in uscfDNA was previously demonstrated by BRcfDNA-Seq and in similar studies (11,14).

In addition, the uscfDNA fragments had the highest enrichment in H3K4me3 and hypomethylated regions compared to controls (random genomic regions) (Figure 2C). These genome regions may have more accessible chromatin organization to nucleases, generating hypomethylated uscfDNA in circulation (60,61). Another study reported that the pattern of cfDNA fragmentation of H3K4me3 resembles the fragmentation pattern of regions of housekeeping genes, in contrast to H3K9me3, which matches repressed genes (62). That report did not include uscfDNA analysis, which may have demonstrated an even more distinct fragment pattern between active and non-active regions of the genome. To confirm these findings, ChIP assays could be performed using plasma to determine if uscfDNA is immunoprecipitated with the proteins assayed. Another intriguing possibility is that DNA secondary structures themselves, such as G-Quads, could confer protection from circulating nucleases. Functionally, G-Quad structures have been associated with open chromatin regions near promoters and with increased transcription through specific recognition by transcription factors (63,64).

Further support for the hypothesis that a subpopulation of uscfDNA can report gene expression activity is that we were able to recapitulate the prior observation that ultrashort fragments are enriched along the TSSs compared with mncfDNA, which has decreased coverage (Figure 5A) (13,45,65). Hypomethylated uscfDNA fragments exhibited increased enrichment through the TSSs of highly expressed hemopoietic genes, whereas the opposite was observed in mncfDNA fragments (Figure 5B). Creative attempts to study the dimension of cfDNA fragmentation patterns to infer gene expression can potentially be applied with uscfDNA. It appears this correlation with expression is most pronounced with the 0% CpG methylation subpopulation of uscfDNA fragments and the > 75 to 100% mncfDNA fragments (Figure 5B and E). Target enrichment of the TSS region may allow for single-gene expression resolution and be another strategy for deconvoluting micro signals within the global activity noise in cfDNA.

Examining the common CpG regions for differential methylation, there were regions in which uscfDNA had higher levels of CpG methylation than mncfDNA. However, the majority of significant DMRs were from regions of decreased methylation in uscfDNA, reflecting the global trend of the two circulating DNA populations. Furthermore, most DMRs were near TSSs, suggesting potential differences in gene regulation related to uscfDNA and mncfDNA sequences (Supplementary Figure S9).

The deconvolution attempt predicted that mncfDNA are derived from an assortment of blood cells that matched with expected cell type levels in the blood (66) and other prior cfDNA studies that used DMRs for deconvolution (10,67) (Figure 6B). The uscfDNA showed significant enrichment in eosinophils, which are reported to exhibit efficient DNA repair machinery for both double-strand and single-strand breaks (68). One possibility is that, because uscfDNA is enriched in simple repeats, which are predisposed to double-strand break damage (69), the efficient repair process in blood cells, such as eosinophils, may lead to the generation of circulating uscfDNA by-products. Eosinophils are also reported to release DNA-based extracellular traps into circulation, which is another potential source of uscfDNA (70,71).

Various CpG-related cfDNA characteristics could be useful biomarkers to differentiate between non-cancer and NSCLC samples. When CpG methylation ratios for each size fragment were considered, the NSCLC samples appeared more hypermethylated in size bins <140 bases (Supplementary Figure S10). This observation contrasted with the genome-wide hypomethylation typically observed in cancer cells compared to healthy cells (6). However, the regions covered by cfDNA, particularly uscfDNA, do not faithfully represent the genome in its entirety, as uscfDNA appears to be enriched in regulatory regions (Figure 1G) (11,13–15). Hypomethylation of transcriptionally active regions seems to occur less frequently in lung cancer (72–74). In addition, cfDNA is composed predominantly of DNA from blood cells rather than cancer tissue exclusively, which can explain the discrepancy.

Cancer-specific promoters and CpG Islands may become hypermethylated (75). In our sample set, both NSCLC uscfDNA and mncfDNA demonstrated substantial hypermethylation in the promoter, 5′UTR, CpG island and exon elements compared to non-cancer samples (Figure 7C and D and Supplementary Figure S11).

In contrast to the other elements, which were either hypermethylated or variable, we observed that the LINEs and SINEs of NSCLC samples trended towards a hypomethylated state. In the genome, LINEs and SINEs have been described to undergo hypomethylation in cancer (72). These high variability traces may indicate micro instability in the epigenetic regulation of these elements. The greater separation in mncfDNA may be due to the greater contribution of tumor-derived fragments, which have been shown to be enriched at 90–150 bases (76). It is unclear whether the changes in methylation patterns originate from an increasing load of tumor-cfDNA or adjustments in immune system activity.

The limited number of DMRs for uscfDNA resulted from the overlap between the two uscfDNA fractions. Regardless, we were able to show that both uscfDNA and mncfDNA bins could be a valuable source of candidate DMRs (Figure 7E). The increased expression of *PK3P* is associated with various types of cancer, including colon, lung, and bladder cancer (77,78). *CPLX1* is one of several factors able to influence the activity of cyclin B1 (*CCNB1*), which is highly expressed in lung adenocarcinoma and associated with poor prognosis (79). *CPLX1* has been documented to promote malignancy in gastric cancer (80). The expression of *COL26A1* has been observed to be downregulated in patients with transformed small-cell lung carcinoma who respond well to PD-L1 inhibitors. For mncfDNA candidates, mutations in *ZNF595* have been indicated as a potential germline mutation in familial lung cancer (81) and a region for prevalent somatic mutations in gastric cancer (82). The non-pseudo gene version of *MLLT10* has been documented to be a promoter of tumor cell proliferation, migration, and invasion in NSCLC cell lines (83), and *MLLT10P1* is commonly mutated in breast cancer patients (84). *NERUDO2* is hypermethylated *in situ* in adenocarcinoma tissues, contrasting the hypomethylation we saw in our study (85). Despite the potential biological rationale, these DMRs are not yet validated. However, this approach shows the merit of DMR discovery, which could give rise to useful targets for future cancer detection.

Surprisingly, the deconvolution prediction did not indicate a signal from lung tissues despite the samples being from NSCLC subjects. Despite late-stage cases, most cfDNA is still of blood cell origin (66). For uscfDNA, the starkest change was a decrease in the percent of eosinophils and a trend in increased neutrophils. Increased eosinophils have been associated with improved prognosis in lung cancer (86,87). In mncfDNA, the megakaryocytes were increased, which has also been described as being associated with cancer in the literature (25,88,89).

Using whole-genome sequencing, other investigators have reported that uscfDNA containing potential G-Quad secondary structures is decreased in cancer patients (13). This pattern was also observed in NSCLC samples after bisulfite conversion (Figure 5E). Interestingly, in NSCLC samples, fragments that contained potential G-Quad structures had increased CpG methylation levels compared to non-cancer samples (Figure 8A and B). Within the genome, G-Quad elements have been described as regulating methylation behavior at CpG Islands (90,91). It is possible that, although there was a decrease in G-Quad structures present in the plasma, it reflects changes in altered CpG methylation and subsequent changes in transcription factors or chromosomal inaccessibility.

Mutations in chromatin-bound proteins frequently occur in cancer (92). We observed that the percent intersection with epigenetic marks was also altered in NSCLC samples, with the greatest decreases in the intersection of H3K27ac and H3K4me3 for both uscfDNA and mncfDNA (Figure 8C). As these two marks are associated with genes with high expression, their decrease in the NSCLC samples in our study may be suggestive of dysregulation in cancer and a potential viable global indicator.

In conclusion, the 5mCAdpBS-Seq single-stranded DNA library preparation is advantageous for uscfDNA methylation profile investigation due to preservation of the native fragment length and methylation level in each size bin. Using this protocol, the methylation characteristics of uscfDNA appear to be distinctly different from those of mncfDNA, further illustrating that it should be considered a separate cfDNA molecule. As a methylation-based cancer biomarker, potentially useful features of uscfDNA are global CpG methylation% changes, genome element profiles, CpG-methylation traces for specific elements, DMRs, tissue-of-origin deconvolution, G-Quad signature changes, and epigenetic mark association. Although we focused on cfDNA from plasma, the 5mCAdpBS-Seq protocol is useful for any context in which very short DNA templates are present. This can include analysis of other biofluids with fragmented DNA (e.g. saliva and urine (93,94)), cell-culture conditioned media environments (95), or theoretically any in-vitro intracellular study in which the accurate methylation analysis of short single-stranded DNA is required. Therefore, if investigators are interested in examining the methylation profile of a DNA sample with heterogeneous sizes, the 5mCAdpBS-Seq protocol should be considered.

## Supplementary Material

gkae276_Supplemental_File

## Data Availability

The sequencing data were deposited in the National Institute of Health Sequence Read Archive under accession number PRJNA980280 and GEO accession number GSE252088. Processing scripts and analysis commands are found in Zenodo at https://doi.org/10.5281/zenodo.10895251.
