# Supplementary material for: Single-stranded pre-methylated 5mC adapters uncover the methylation profile of plasma ultrashort Single-stranded cell-free DNA
Source: Nucleic Acids Res. 2024 May 27;52(11):e50. doi: 10.1093/nar/gkae276 (PMC11194076; doi:10.1093/nar/gkae276)
Supplement: gkae276_Supplemental_File [file gkae276_supplemental_file.pdf]

SUPPLEMENTARY DATA

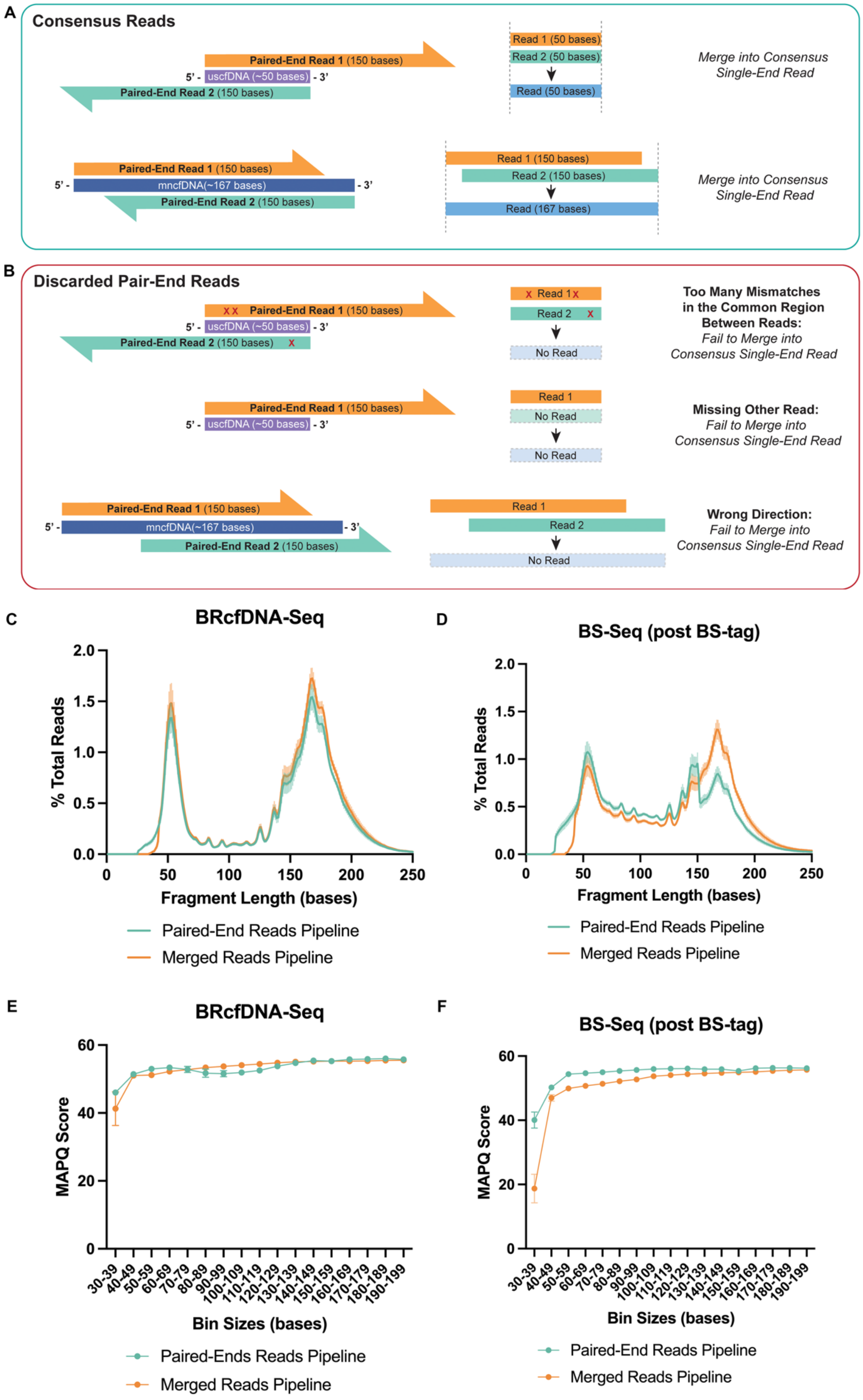
A
Consensus Reads
Paired-End Read 1 (150 bases)
5' - uscfDNA (~50 bases) - 3'
Paired-End Read 2 (150 bases)
Read 1 (50 bases)
Read 2 (50 bases)
Read (50 bases)
Merge into Consensus Single-End Read
Paired-End Read 1 (150 bases)
5' - mncfDNA(~167 bases) - 3'
Paired-End Read 2 (150 bases)
Read 1 (150 bases)
Read 2 (150 bases)
Read (167 bases)
Merge into Consensus Single-End Read
B
Discarded Pair-End Reads
X X Paired-End Read 1 (150 bases)
5' - uscfDNA (~50 bases) - 3'
Paired-End Read 2 (150 bases) X
X Read 1 X
Read 2 X
No Read
Too Many Mismatches in the Common Region Between Reads:
Fail to Merge into Consensus Single-End Read
Paired-End Read 1 (150 bases)
5' - uscfDNA (~50 bases) - 3'
Read 1
No Read
No Read
Missing Other Read:
Fail to Merge into Consensus Single-End Read
Paired-End Read 1 (150 bases)
5' - mncfDNA(~167 bases) - 3'
Paired-End Read 2 (150 bases)
Read 1
Read 2
No Read
Wrong Direction:
Fail to Merge into Consensus Single-End Read
C
BRcfDNA-Seq
% Total Reads
Fragment Length (bases)
Paired-End Reads Pipeline
Merged Reads Pipeline
D
BS-Seq (post BS-tag)
% Total Reads
Fragment Length (bases)
Paired-End Reads Pipeline
Merged Reads Pipeline
E
BRcfDNA-Seq
MAPQ Score
Bin Sizes (bases)
Paired-Ends Reads Pipeline
Merged Reads Pipeline
F
BS-Seq (post BS-tag)
MAPQ Score
Bin Sizes (bases)
Paired-End Reads Pipeline
Merged Reads Pipeline

Supplementary Figure 1. Merging paired-end reads results in a similar profile to untreated non-targeted sequencing. The schematic demonstrates pre-merging pipeline paired reads prior to alignment. **(A)** Situations in which reads are accepted for downstream analysis. With uscfDNA (~50 bases), the consensus sequence of reads 1 and 2 should have 100% overlap, whereas for mncfDNA (~167 bases), there will be a 150-base perfect overlap of reads 1 and 2, with 17 bases with no overlap. These reads will still be accepted. **(B)** Potential scenarios in which reads fail to merge and are discarded from downstream analysis. (**C)** BRcfDNA-Seq libraries show little difference in the pattern with and without merging via the processing pipeline. **(D)** BS-Seq libraries show the difference in the pattern when reads are merged prior to alignment (dip at 150 bases). MAPQ scores for binned reads of 10 bases for BRcfDNA-Seq **(E)** and BS-Seq **(F)** libraries for both paired ends and merged bioinformatics preprocessing. In (C) and (D), vertical lines indicate SEM obtained for five samples. Some error bars may not be observable due to their length being smaller than the size of the data point.

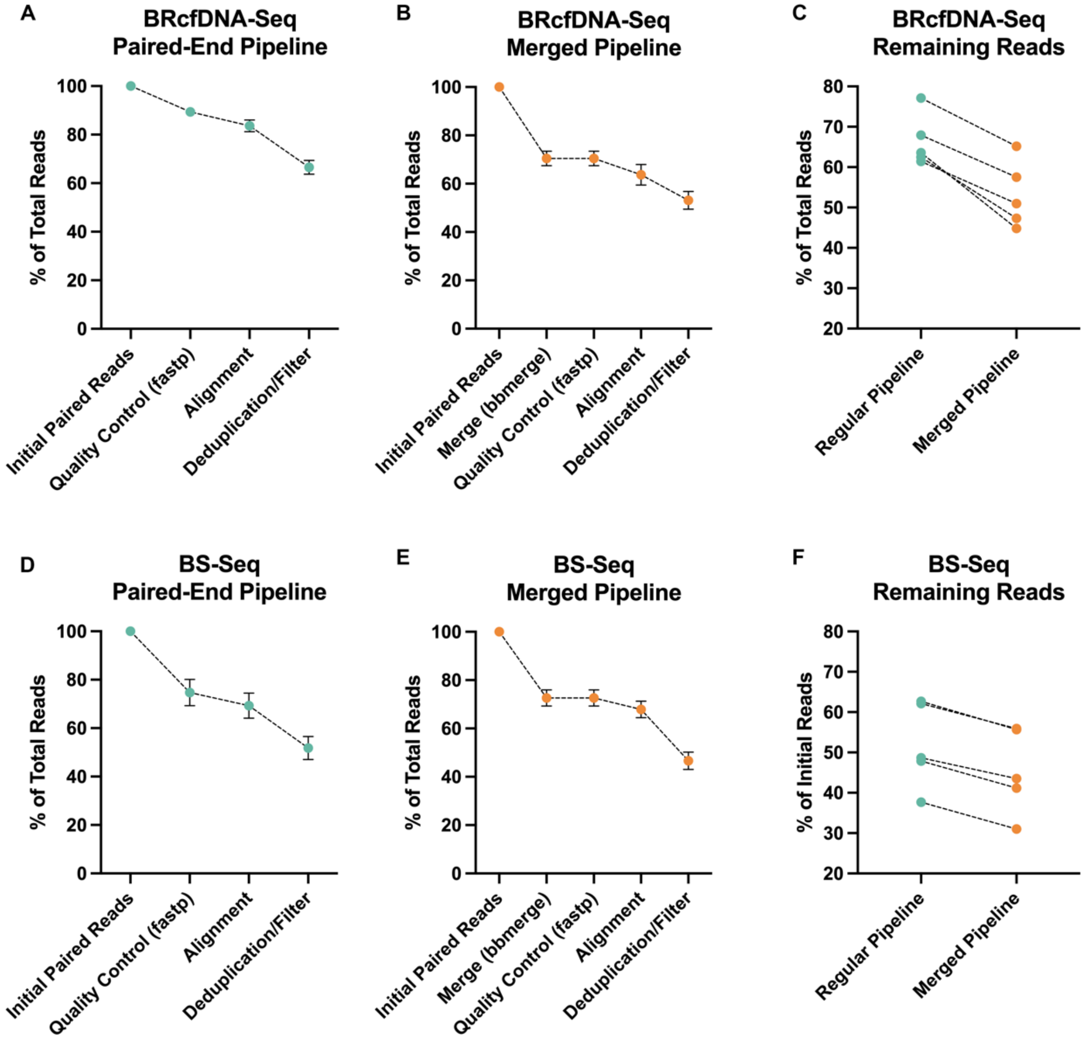

Supplementary Figure 2. The majority of reads are excluded during the initial merging of reads. (**A**) The percent of total reads is shown after each bioinformatics preprocessing step comparing BRcfDNA-Seq libraries processed using both the paired-end reads or **(B)** merged reads protocol, and (**D**) BS-Seq libraries using paired-end or **(E)** merged read processing. (**C**) A comparison of final read count of individual samples between paired-end read vs. merged read pipelines for BRcfDNA-Seq and **(F**) BS-Seq, Error bars indicate SEM.

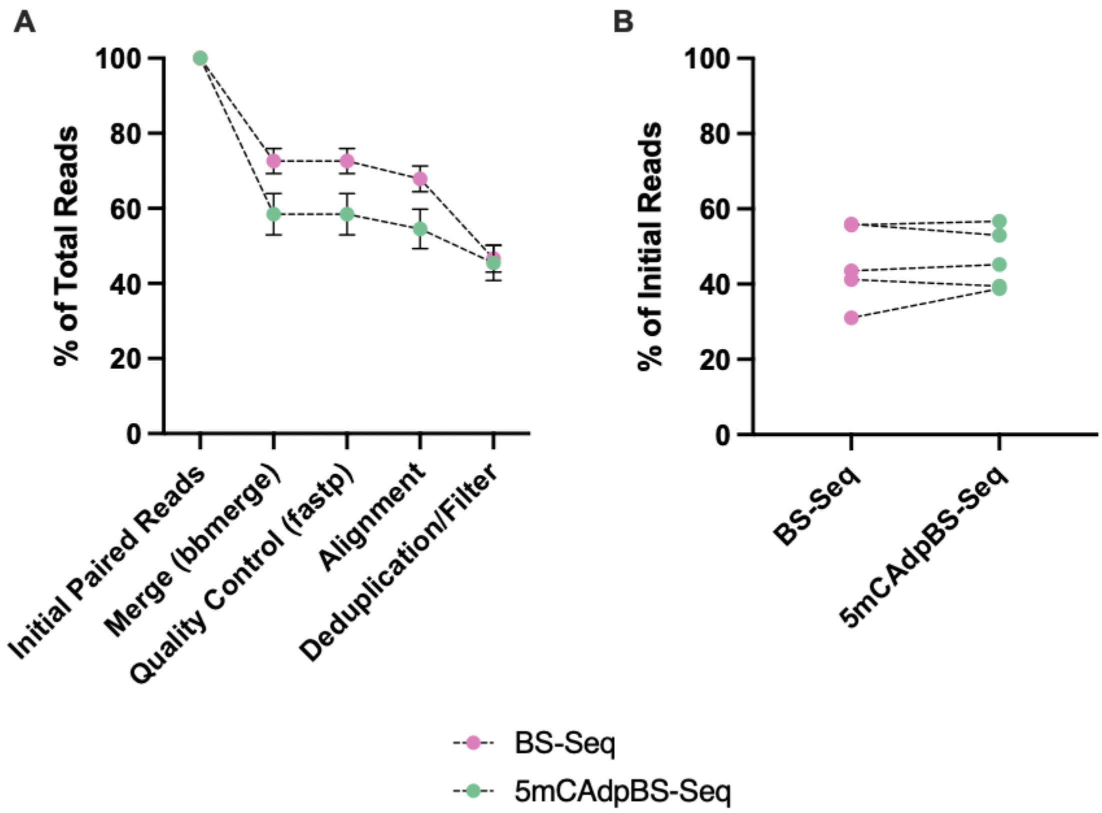

Supplementary Figure 3. Comparative read attrition between BS-Seq and BS-Seq (5-mC Adp) during bioinformatics processing. **(A)** Comparison of BS-Seq vs. 5mCAdpBS-Seq protocols in regard to the read attrition during each step of the preprocessing pipeline prior to downstream analysis. (**B)** Final remaining reads for five individual plasma samples that underwent each protocol.

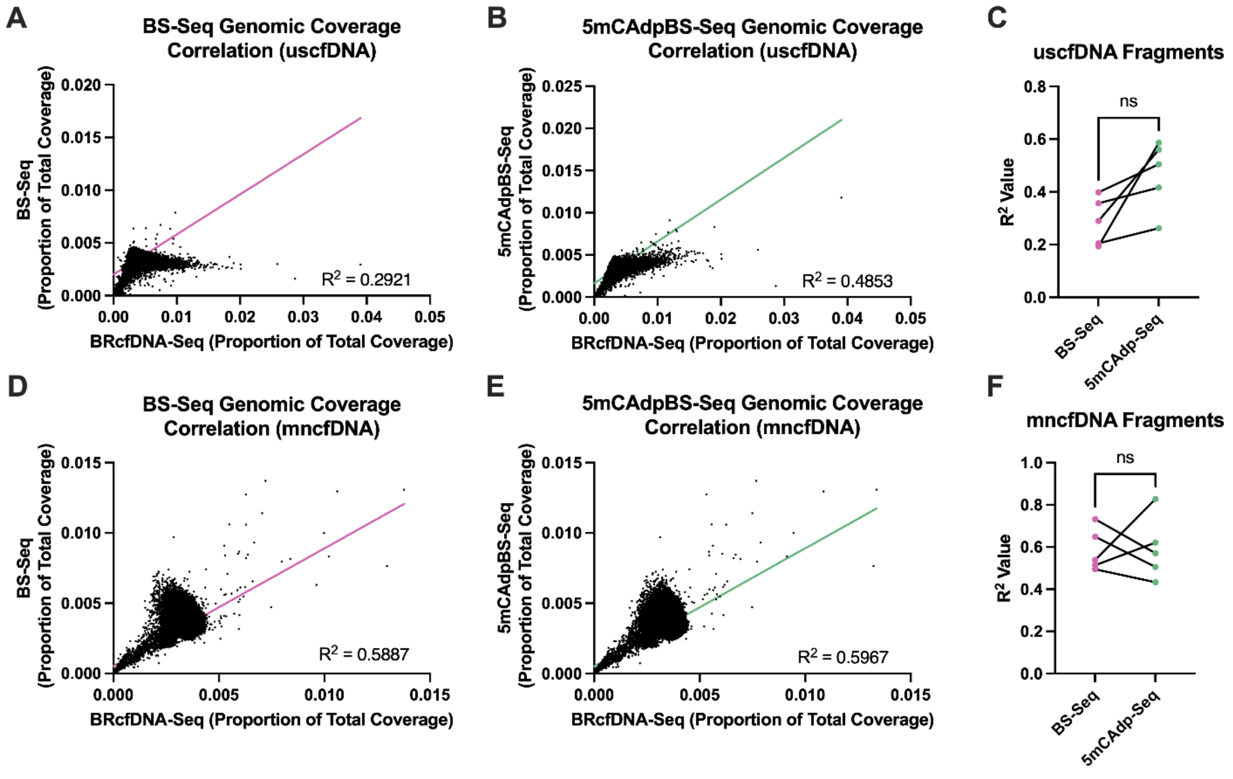

Supplementary Figure 4. Linear correlation of genomic coverage for BS-Seq and 5mCAdPBS-Seq compared to BRcfDNA-Seq for uscfDNA (**A and B**) and mncfDNA (**D and E**). For uscfDNA fragments, the linear correlation coefficient is higher for samples processed with the 5mCAdpBS-Seq protocol than those prepared for the BS-Seq. Data present the average of five samples. Paired t-test for uscfDNA (C) and mncfDNA (F) fragments showed, on average, a higher R^2 value for uscfDNA fragments processed by the 5mCAdpBS-Seq protocol.

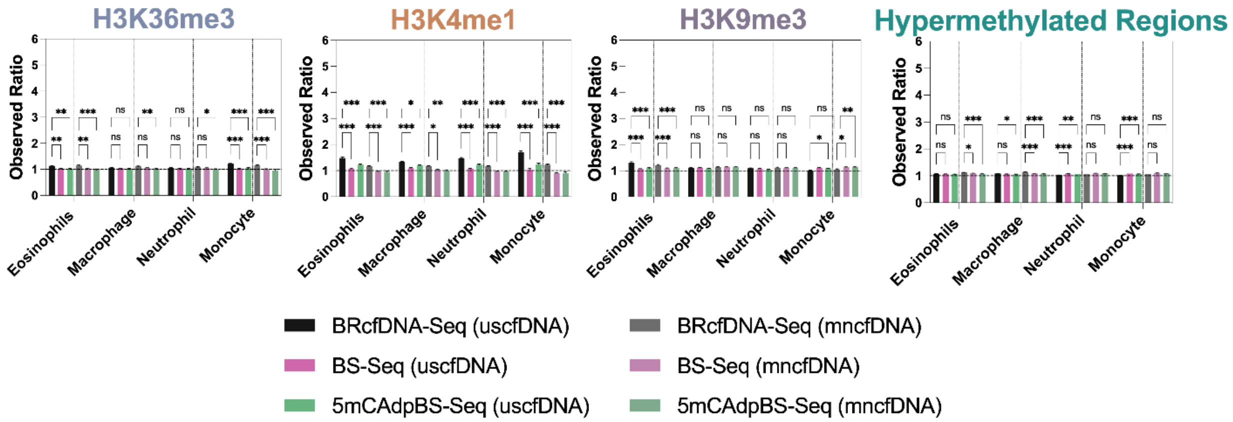

Supplementary Figure 5. Observed ratio of intersecting bases in the bed file to intersecting bases in randomly shuffled control files for other epigenetic marks not shown in the main figure for uscfDNA and mncfDNA bins. Randomly shuffled bed files were generated for each sample to act as a control for intersection locations. The horizontal dotted line represents the observed ratio of 1.0. Data are presented as the mean and SEM of five paired non-cancer samples. * $p<0.05$, ** $p<0.01$, *** $p<0.001$, Tukey's multiple comparison test after two-way ANOVA. Only comparisons with BRcfDNA-Seq are shown.

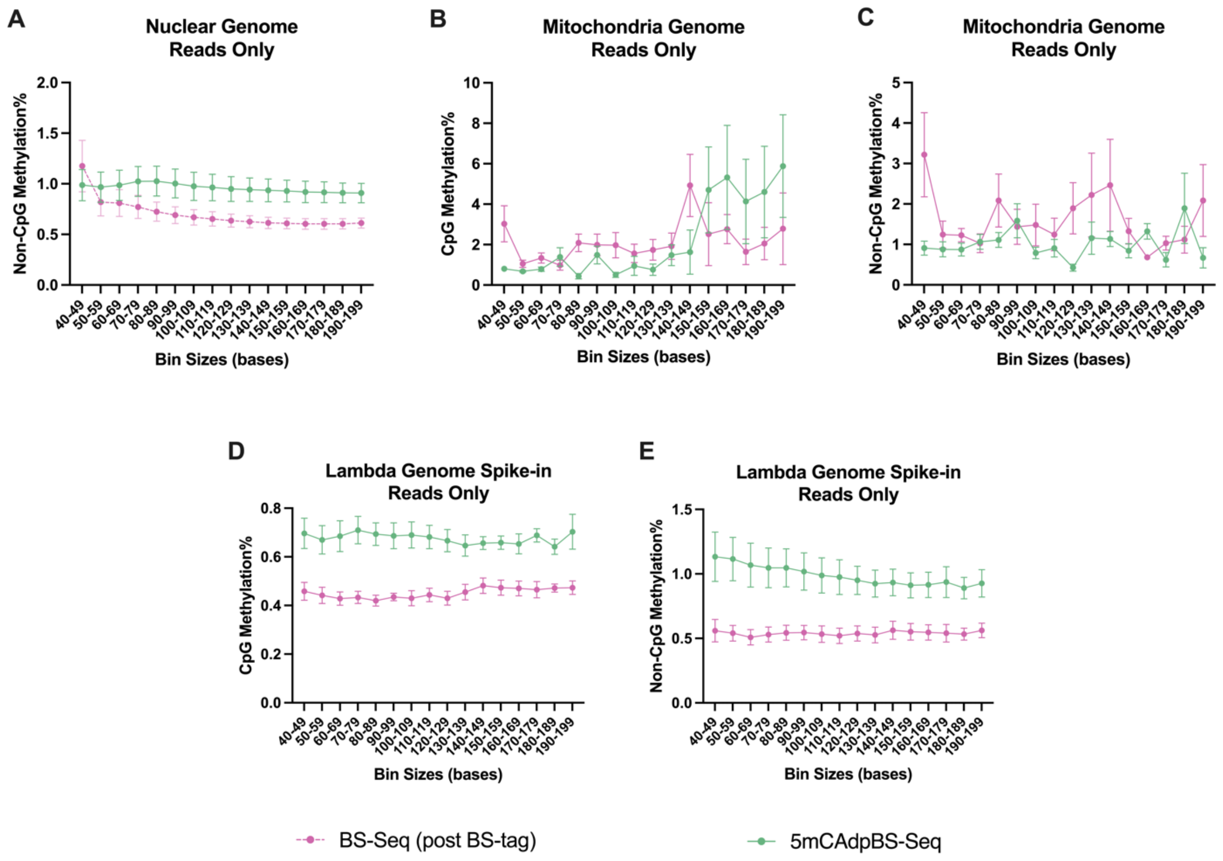

Supplementary Figure 6. (A) Zoom-in scale for low CpG and non-CpG methylation bins in nuclear, **(B, C)** mitochondrial**,** and (**D, E**) lambda spike-in reads. Samples are from five paired samples undergoing both protocols. Vertical lines and error bars indicate the mean and SEM from five samples.

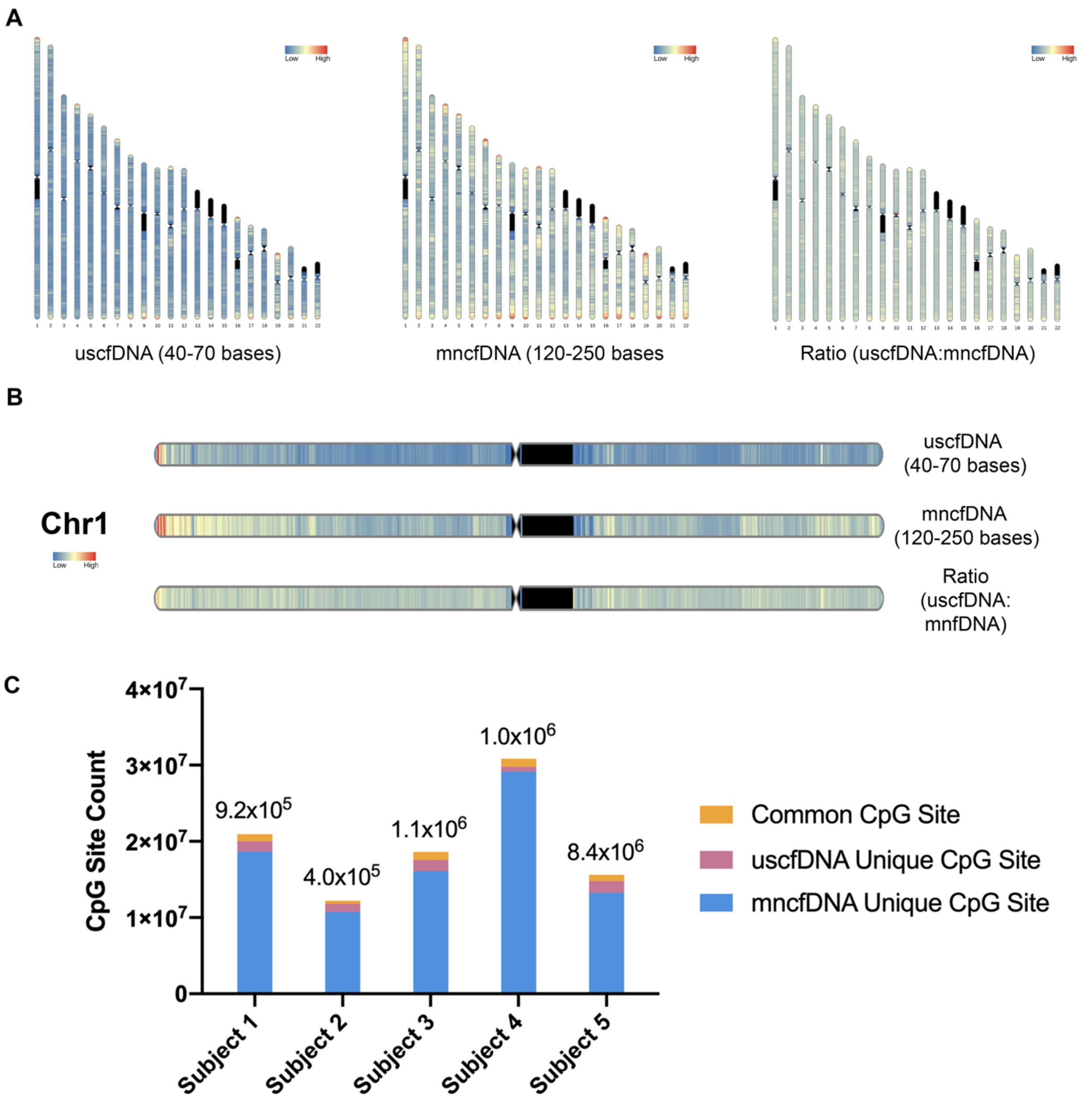

Supplementary Figure 7. CpG positions differ in uscfDNA and mncfDNA. Karyograms averaged from five non-cancer samples regarding the % coverage for 1 million base-sized bins across all chromosomes **(A)** and chromosome 1 **(B)**. The ratio was calculated by dividing the mean uscfDNA %coverage by mncfDNA %coverage. **(C)** Intra-sample count of common and unique CpG site counts between cfDNA populations. Values above bars indicate the count of common CpG sites. Data are presented as the mean and SEM of five paired non-cancer samples processed with 5mcAdpBS-Seq. * p<0.05, ** p<0.01 , *** p<0.001, and **** p<0.0001 (unadjusted).

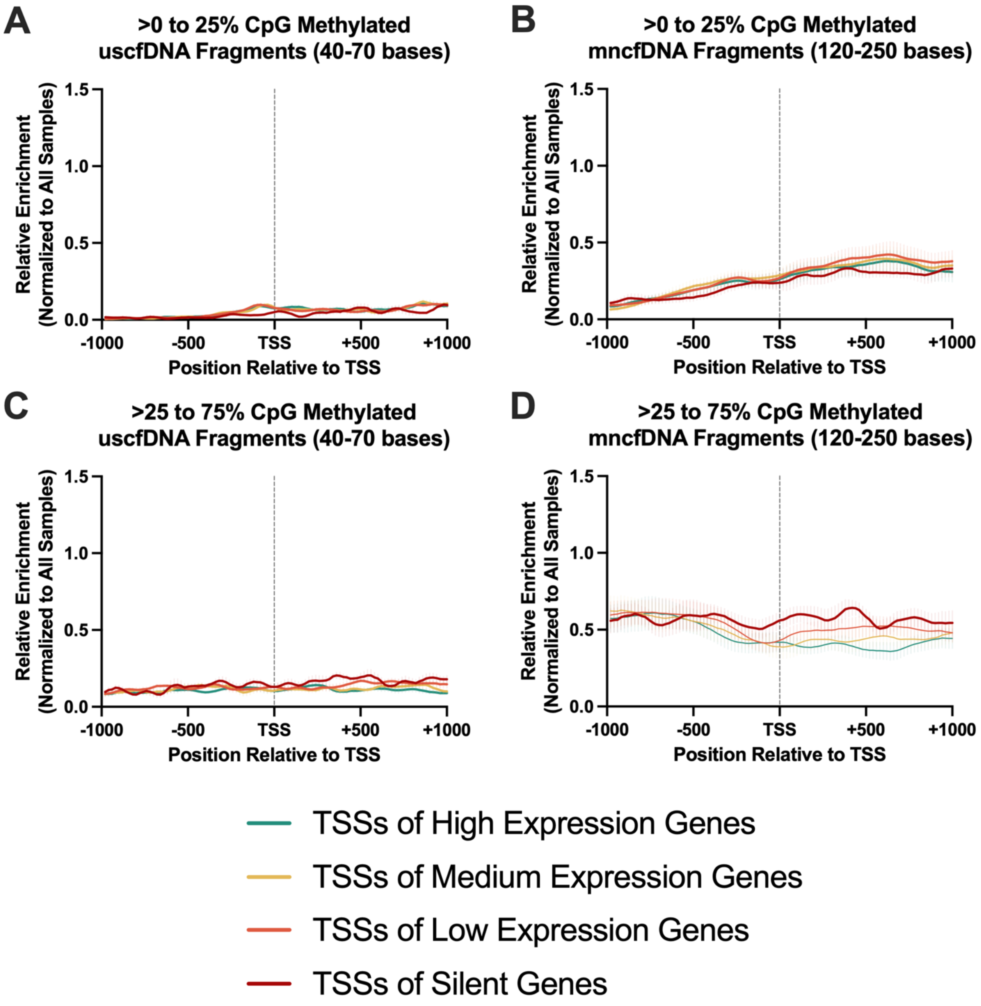

Supplementary Figure 8. Pattern of enrichment of CpG fragments -1000 bases upstream and +1000 bases downstream from the transcription start site for differentially expressed genes in uscfDNA and mncfDNA fragments. (**A, B**) With ≤24% CpG methylation and (**C, D**) 25-74% CpG methylation. TSS categories were based on the RNA expression activity from RNA-Seq experiments with the buffy coat from previous literature. High expression was considered >41.07 RPKM, medium 15.36-41.06 RPKM, low 1-15.36 RPKM, and silent <0 RPKM. Lines show five separate non-cancer samples processed with the 5mCAdpBS-Seq protocol. Enrichment was normalized to all samples in the comparison.

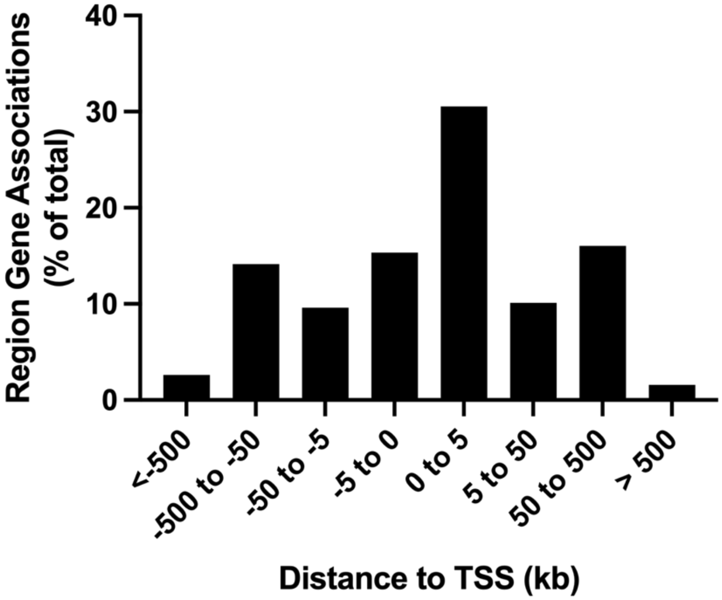

Supplementary Figure 9. The majority of DMRs between uscfDNA and mncfDNA are in close vicinity to TSS. Only DMR candidates with q-value <1.0 from five samples between merged uscfDNA and mncfDNA .bam files are shown. The total count was 573.

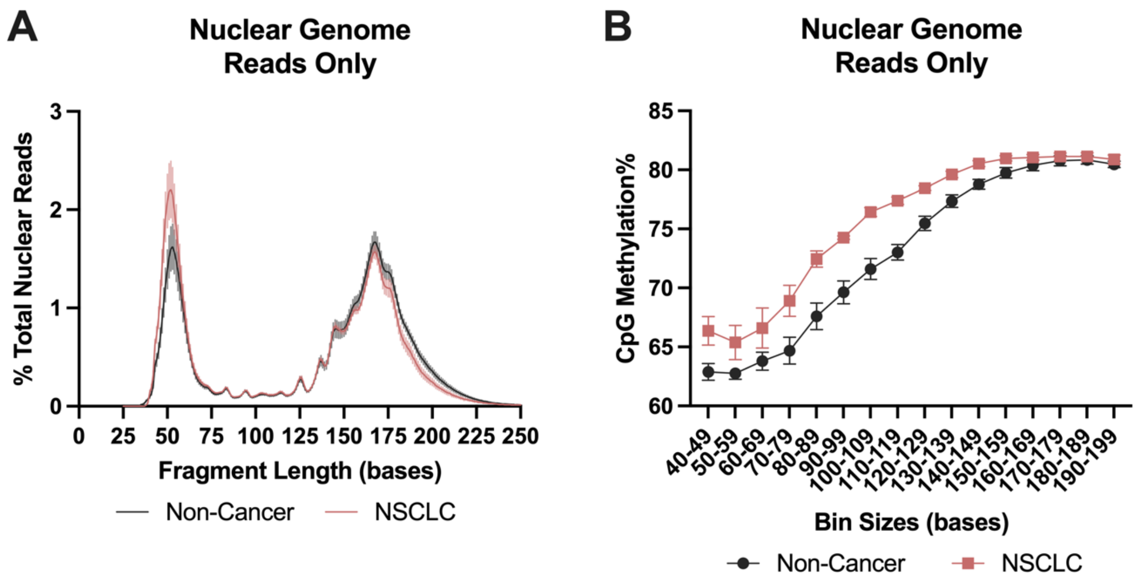

Supplementary Figure 10. Genomic and methylation profiles differ between non-cancer and NSCLC samples processed by 5mCAdpBS-Seq. **(A)** Fragment size distribution profile comparing non-cancer and NSCLC cohorts. (**B)** CpG methylation as % of uscfDNA region was elevated in non-small cell lung carcinoma samples compared to non-cancer samples. Plots represent the mean and SEM of five paired non-cancer plasma and four NSCLC samples with the 5mCAdpBS-Seq protocol.

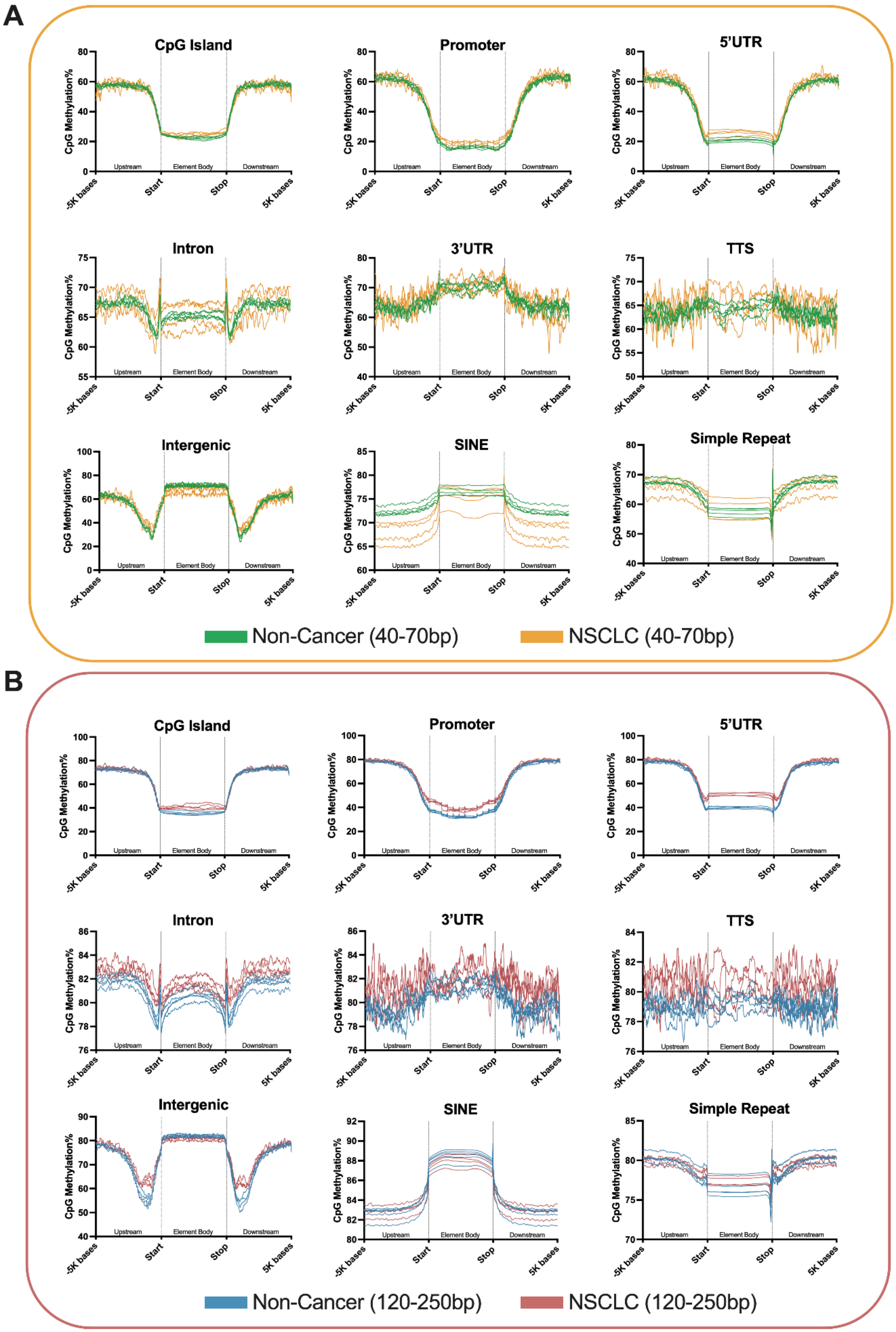
A
CpG Island
Promoter
5'UTR
Intron
3'UTR
TTS
Intergenic
SINE
Simple Repeat
CpG Methylation%
Upstream
Element Body
Downstream
-5K bases
Start
Stop
5K bases
Non-Cancer (40-70bp)
NSCLC (40-70bp)
B
CpG Island
Promoter
5'UTR
Intron
3'UTR
TTS
Intergenic
SINE
Simple Repeat
CpG Methylation%
Upstream
Element Body
Downstream
-5K bases
Start
Stop
5K bases
Non-Cancer (120-250bp)
NSCLC (120-250bp)

Supplementary Figure 11. CpG methylation patterns differ between non-cancer and NSCLC samples. (**A**) The average CpG methylation % patterns from 5000 bases upstream and 5000 bases downstream are plotted for each genomic element for uscfDNA and (**B**) mncfDNA-sized reads. SINE: short interspersed nuclear element, LINE: long interspersed nuclear element, TTS: transcription termination site, 5'UTR: 5' untranslated region, 3'UTR: 3' untranslated region. Lines show five paired plasma samples that underwent the 5mCAdpBS-Seq protocol.

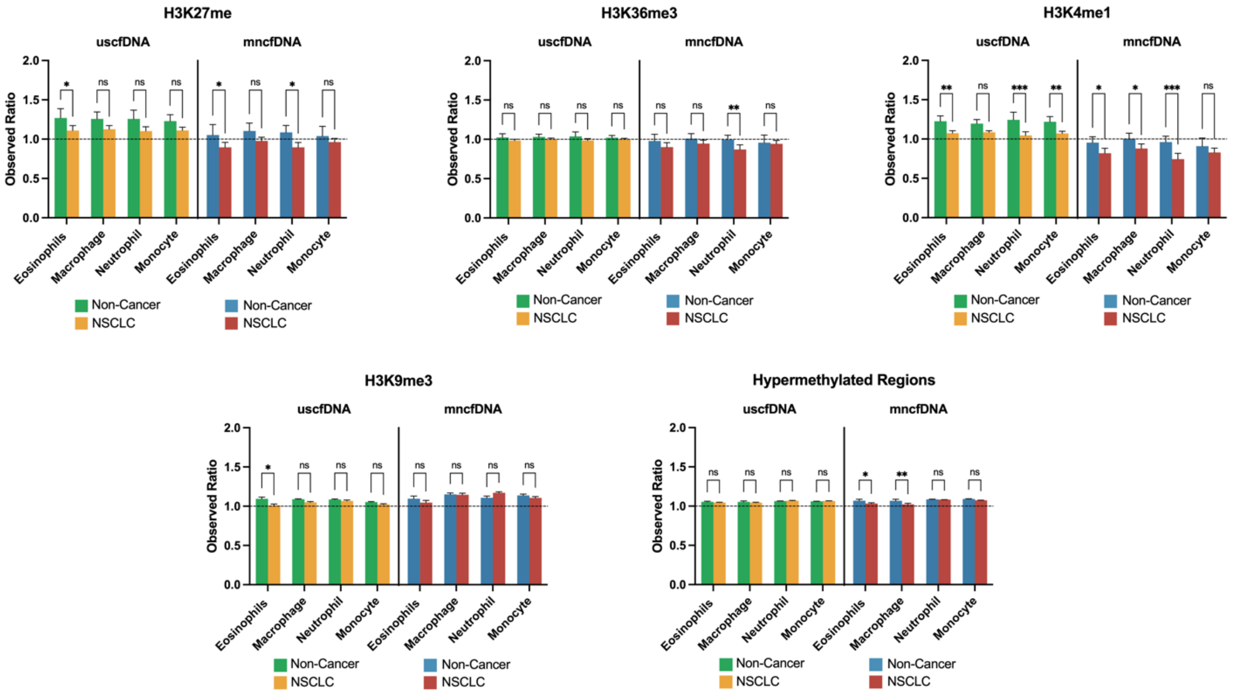

Supplementary Figure 12. Normalized percentage of intersecting bases for epigenetic marks H3K27me, H3K36me3, H3K5me1, H3k9me3, and hypermethylated regions. Minor changes were observed between NSCLC samples and non-cancer samples in both uscfDNA and mncfDNA bins. The % intersection was normalized to control shuffled bed files. The horizontal dotted line represents the observed ratio of 1.0. Data are presented as the mean and SEM of five paired non-cancer samples and four NSCLC plasma samples. * $p<0.05$, ** $p<0.01$, *** $p<0.001$, Tukey's multiple comparison test after two-way ANOVA.

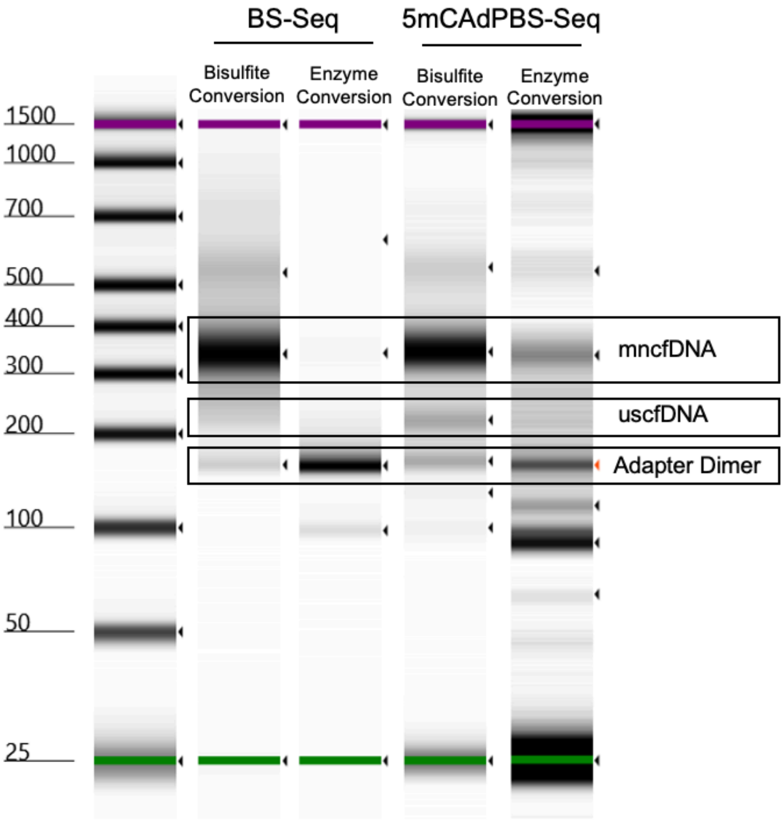

Supplementary Figure 13. Enzymatic conversion of 5mC did not generate sufficient libraries. Electrophoresis gel of the comparison of bisulfite and enzyme conversion protocols for extracted cell-free DNA from 2 mL of non-cancer plasma after single-stranded library preparation. BS-Seq and 5mCAdpBS-Seq protocols are shown. The enzyme conversion protocol generates libraries with only adapter dimers or cell-free DNA-sized bands with low concentrations.

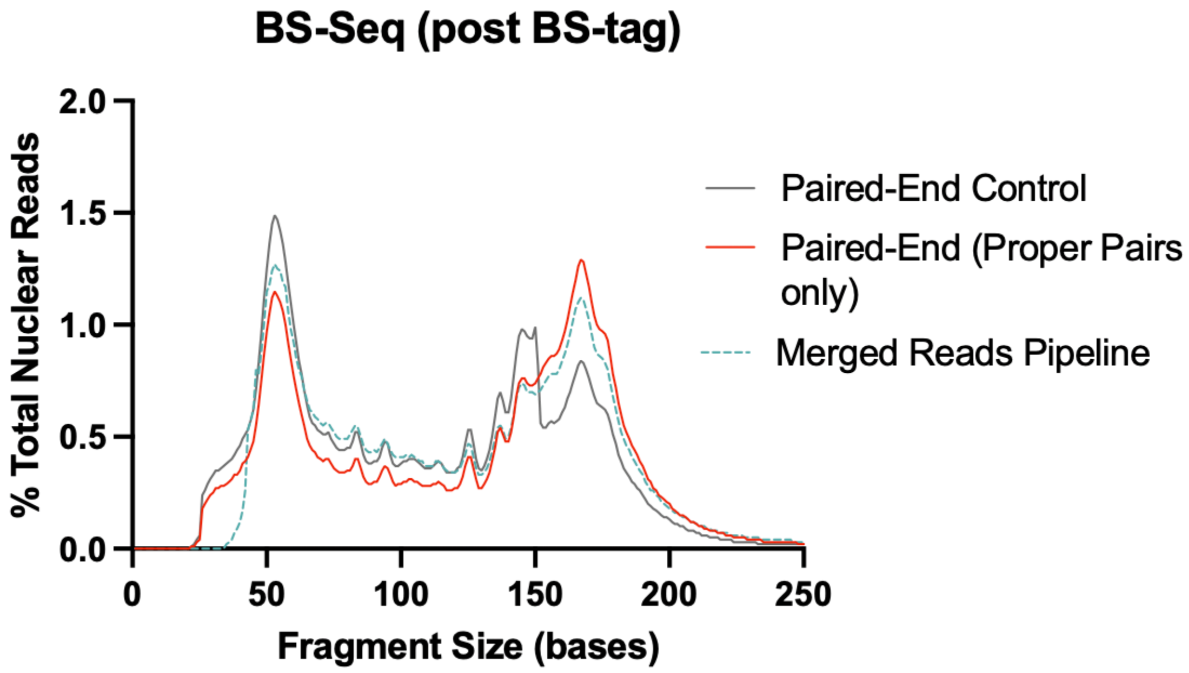

Supplementary Figure 14. Filtering for proper pairs (R1 and R2 orientated towards each other), the paired-end reads pipeline mimics the size distribution curve of the merged reads pipeline.
